# Isoprenylated flavonoids and clerodane diterpenoids from *Dodonaea viscosa*

**DOI:** 10.1007/s13659-013-0053-4

**Published:** 2013-10-16

**Authors:** Yuan Gao, Yin-Dong Fang, Ping Hai, Fei Wang, Ji-Kai Liu

**Affiliations:** 1 State Key Laboratory of Phytochemistry and Plant Resources in West China, Kunming Institute of Botany, Chinese Academy of Sciences, Kunming, 650201 China; 2 BioBioPha Co., Ltd., Kunming, 650201 China; 3 University of Chinese Academy of Sciences, Beijing, 100049 China

**Keywords:** *Dodonaea viscosa*, isoprenylated flavonoid, clerodane diterpenoid, dodovisone, dodovislactone

## Abstract

Phytochemical investigation of the aerial parts of *Dodonaea viscosa* led to the isolation of six new compounds including four isoprenylated flavonoids, dodovisones A-D (**1**–**4**), and two clerodane diterpenoids, dodovislactones A and B (**5** and **6**). Their structures were established by extensive spectroscopic analysis.

**Electronic Supplementary Material:**

Supplementary material is available for this article at 10.1007/s13659-013-0053-4 and is accessible for authorized users.

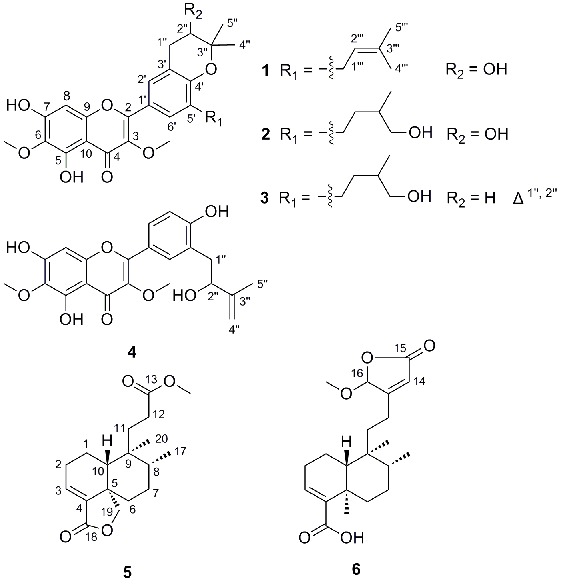

## Introduction

The genus *Dodonaea* (Sapindaceae) is composed of approximately 50 species and is mainly distributed throughout Australia and its nearby islands. Only one species exists in China, *Dodonaea viscosa*; a eurytopic species growing throughout the tropics and sub-tropics.[1] This plant has been used as a folk medicine for the treatment of fever, skin diseases, stranguria, toothache, rheumatism, gout, inflammation, and swelling.[2,3] Previous chemical investigations of *D. viscosa* found flavonoids, diterpenoids, triterpenoid saponins, cyanolipids, and phenylpropanoids, among which isoprenylated flavonoids and clerodane diterpenoids predominated.[2–7] Pharmacological studies on the title plant have shown bioactivities such as antibacterial[8] and antioxidant[8] activities, inhibition against urease[9] and enoyl-ACP reductase,[10] and activity against liver fibrosis.[11] Hou et al. reported that some isoprenylated flavonoids from this plant enhanced the accumulation of lipid droplets significantly and induced the up-regulation of the expression of the adipocyte-specific genes *aP2* and *GLUT4*.[2] Recent pharmacological research on hautriwaic acid and related terpenes derived from this plant also displayed potent inhibitions against edema-associated inflammation which were similar or higher than those of reference compound indomethacin when evaluated in the chronic test.[12]

4

5

6

As part of our effort to assemble a large-scale natural compound library of thousands of structures as well as to examine opportunities for the development of natural resources,[13] the study described herein was undertaken to determine the chemical constituents in the aerial parts of *D. viscosa*. The resulting investigation characterized four isoprenylated flavonoids, dodovisones A–D (**1**–**4**), as well as two clerodane diterpenoids, dodovislactones A and B (**5** and **6**), together with 12 known compounds: dodoviscin J,[2] dodoviscin A,[2] dodoviscin I,[2] dodoviscin H,[2] aliarin,[14] 5,7,4'-trihydroxy-3,6-dimethoxy-3'-prenylflavone,[15] 5'-prenylaliarin,[3] 5,7,4'-trihydroxy-3,6-dimethoxyflavone,[16] sakuranetin,[17] 15-methoxypatagonic acid,[18] 6α-hydroxycleroda-3,13-dien-16,15-

*To whom correspondence should be addressed. E-mail: jkliu@mail.kib.ac.cn olid-18-oic acid;[19] and hautriwaic acid.[20] Herein, we describe the isolation and structure elucidation of the new compounds.

## Results and Discussion

Compound **1** was isolated as a yellowish powder with a molecular formula of $C_{27}H_{30}O_8$, as evidenced by HREIMS at *m*/*z* 482.1960 (calcd 482.1941) and NMR spectroscopic data, with 13 degrees of unsaturation. The IR spectrum suggested the presence of hydroxy (3417 $cm^{-1}$) and conjugated carbonyl (1656 $cm^{-1}$) groups in **1**. The UV spectrum (MeOH) of **1** showed maxima at 251, 271, and 352 nm suggestive of a flavone nucleus and also closely reflecting the UV absorbances of related flavones isolated from the same species[2]. The $^1H$ NMR spectrum (Table 1) displayed a chelated phenolic hydroxy group at $\delta_H$ 12.94 (1H, br, s), an aromatic proton at $\delta_H$ 6.55 (1H, s), two *meta*-coupled aromatic protons at $\delta_H$ 7.66 and 7.76 (each 1H, d, $J$ = 1.7 Hz), and two methoxy groups at $\delta_H$ 3.83 and 4.03 (each 3H, s). The $^1H$ NMR data also exhibited two isoprenoid units: a 2,2-dimethyl-3-hydroxy-dihydropyran ring[21] [$\delta_H$ 1.41 and 1.36 (each 3H, s), 3.88 (1H, dd, $J$ = 5.5, 4.8 Hz), 2.87 (1H, dd, $J$ = 16.7, 5.5 Hz), and 3.16 (1H, dd, $J$ = 16.7, 4.8 Hz)] ; and a 3-methyl-2-butenyl group[2] [$\delta_H$ 1.74 and 1.76 (each 3H, s), 5.31 (1H, t, $J$ = 7.5 Hz), and 3.36 and 3.32 (each 1H, dd, $J$ = 15.5, 7.5 Hz)]. The $^{13}C$ NMR (Table 1) spectrum showed 27 carbon signals corresponding to a flavonol derivative with two isoprenoid moieties and two methoxy groups. The presence of the dihydropyran ring was further supported by the HMBC correlations (Figure 1) from H-1″ to C-2″ and C-3″, and from H-5″ to C-2″, C-3″, and C-4″. Given that no correlation was observed for H-2″ to C-4′, weak correlation from $H_3$-4″ to C-4a ($^4J$) strongly suggested that an oxygen atom was to bridge C-4′ ($\delta_C$ 153.2) and C-3″ ($\delta_C$ 77.2) and not C-4′ and C-2″. This unit was fused to ring B as shown via C-3′ and C-4′ by the HMBC correlations from H-1″ to C-2′, C-3′, and C-4′. Another isoprenoid unit was confirmed by HMBC correlations from H-2‴ to C-1‴, C-4‴, and C-5‴, and from H-1‴ to C-2‴ and C-3‴ and was attached to C-5′ by correlations from $H_2$-1‴ to C-4′, C-5′, and C-6′. During our work to elucidate hundreds of structures of aromatic methoxy-containing compounds, we found that the particular low field chemical shifts of the aromatic methoxy carbon signals at about 60 ppm (aromatic methoxy carbon signals were commonly at about 56 ppm), were without exception accompanied by *o*-substitutions on both sides of the methoxy groups.[22] This observation may be explained by the steric hindrance of neighboring groups within the same plane. According to this regulation, the two methoxy groups ($\delta_H$ 3.83; $\delta_C$ 60.1/$\delta_H$ 4.03; $\delta_C$ 60.9) were assigned at C-3/C-6, since a chelated phenolic hydroxy group at $\delta_H$ 12.94 (1H, br. s) must be connected to C-5 and the two low field *meta*-coupled aromatic protons at $\delta_H$ 7.66 and 7.76 (each 1H, d, $J$ = 1.7 Hz) can easily be assigned as H-2′/H-6′. This deduction was further supported by the HMBC correlations from $\delta_H$ 3.83 to C-3 ($\delta_C$ 138.2) and from $\delta_H$ 4.03 to C-6 ($\delta_C$ 129.9). The HMBC correlations from the aromatic singlet at $\delta_H$ 6.55 to five quaternary carbons C-6, C-7, C-9, C-10, and C-4 ($^4J$, ω-coupled) indicated that the singlet was assigned to H-8. Accordingly, the hydroxy group leftover must be attached to C-7 ($\delta_C$ 154.8). On the basis of the above discussion, the structure of **1** was finally assigned as 2-[3,4-dihydro-3-hydroxy-8-(3-methylbut-2-en-1-yl)-2,2-dimethyl-2*H*-1-benzopyran-6-yl]-5,7-dihydroxy-3,6-dimethoxy-4*H*-1-benzopyran-4-one, and it was given the trivial name dodovisone A.

**Figure 1.** Key HMBC correlations of **1**

Compound **2** was isolated as a yellowish powder with the molecular formula $C_{27}H_{32}O_9$, as determined by HREIMS at *m*/*z* 500.2034 $[M]^+$ (calcd 500.2046). The $^1H$ and $^{13}C$ NMR data (Table 1) of **2** indicated this compound to be an isoprenylated flavonoid, similar to **1**. The only difference between them was the 3-methyl-2-butenyl group in **1** changing into a 4-hydroxy-3-methylbutyl group[2] [$\delta_H$ 1.37, 1.60, 1.73, 2.58, and 2.69 (each 1H, m), 3.37 (1H, dd, $J$ = 10.6, 6.6 Hz), 3.46 (1H, dd, $J$ = 10.6, 5.9 Hz), and 0.99 (3H, d, $J$ = 6.7 Hz); $\delta_C$ 28.8 ($CH_2$), 34.6 ($CH_2$), 36.2 (CH), 68.4 ($CH_2$), and 17.1 ($CH_3$)] in **2**. This group was confirmed by HMBC correlations from H-5‴ to C-2‴, C-3‴, and C-4‴, and from H-1‴ to C-2‴ and C-3‴. The methylene protons ($\delta_H$ 2.69 and 2.58) of the isoprenoid side chain showed HMBC correlations to C-4′, C-5′, and C-6′, positioning this group at C-5′ as in **1**. Hence, compound **2** was elucidated as 2-[3,4-dihydro-3-hydroxy-8-(4-hydroxy-3-methylbutyl)-2,2-dimethyl-2*H*-1-benzopyran-6-yl]-5,7-dihydroxy-3,6-dimethoxy-4*H*-1-benzopyran-4-one, and it was given the name dodovisone B.

Compound **3** gave a molecular formula of $C_{27}H_{30}O_8$ by HREIMS. Comparison of its $^{13}C$ NMR data (Table 1) with those of **2** revealed similarities, except for the evident methine signals of a double bond ($\delta_C$ 122.1 and 130.8) in **3** instead of a methylene ($\delta_C$ 32.3, C-1″) and an oxygenated methane ($\delta_C$ 70.1, C-2″) in **2**, which was supported by HMBC correlations from H-1″ ($\delta_H$ 6.39) to C-2′, C-3′, and C-4′ and from H-2″ ($\delta_H$ 5.69) to C-3′, C-1″, C-3″, C-4″, and C-5″. Consequently, compound **3** was elucidated as 2-[8-(4-hydroxy-3-methylbutyl)-2,2-dimethyl-2*H*-1-benzopyran-6-yl]-5,7-dihydroxy-3,6-dimethoxy-4*H*-1-benzopyran-4-one and was named dodovisone C.

Compound **4** was assigned the molecular formula $C_{22}H_{22}O_8$ by HREIMS. It was found to be an isoprenylated flavonol with the same ring A and C moieties as **1**–**3**. The ABX spin system observed for aromatic protons in the $^1H$ NMR (Table 1) at $\delta_H$ 7.88 (1H, d, $J$ = 2.2 Hz), 6.88 (1H, d, $J$ = 8.5 Hz), and 7.82 (1H, dd, $J$ = 8.5, 2.2 Hz) suggested a 3′,4′-disubstitution pattern in ring B. A 2-hydroxy-3-methyl-3-butenyl unit[2] was indicated by $^1H$ NMR signals at $\delta_H$ 2.83 (1H, dd, $J$ = 13.7, 7.8 Hz), 2.96 (1H, dd, $J$ = 13.7, 5.2 Hz), 4.39 (1H, dd, $J$ = 7.8, 5.2 Hz), 4.77 (1H, s), 4.87 (1H, s), and 1.81 (3H, s), and another hydroxy group was implied by the presence of an $sp^2$ quaternary carbon at $\delta_C$ 160.1. This butenyl unit was confirmed by HMBC correlations from H-5‴ to C-2‴, C-3‴, and C-4‴, and from H-1‴ to C-2‴ and C-3‴. The above two groups were fixed at C-3′ and C-4′, respectively, by strong

**Table 1. NMR spectroscopic data for 1–4**

| | **1**[a] | | **2**[b] | | **3**[a] | | **4**[b] | |
|---|---|---|---|---|---|---|---|---|
| no. | $\delta_H$ (*J* in Hz) | $\delta_C$ | $\delta_H$ (*J* in Hz) | $\delta_C$ | $\delta_H$ (*J* in Hz) | $\delta_C$ | $\delta_H$ (*J* in Hz) | $\delta_C$ |
| 2 | | 156.6, C | | 158.1, C | | 156.4, C | | 158.4, C |
| 3 | | 138.2, C | | 139.2, C | | 138.2, C | | 139.2, C |
| 4 | | 179.1, C | | 180.1, C | | 179.0, C | | 180.3, C |
| 5 | | 151.7, C | | 153.6, C[c] | | 151.7, C | | 153.7, C[c] |
| 6 | | 129.9, C | | 132.5, C | | 129.8, C | | 132.6, C |
| 7 | | 154.8, C | | 158.6, C | | 154.8, C | | 158.7, C |
| 8 | 6.55, s | 93.0, CH | 6.44, s | 95.0, CH | 6.57, s | 93.0, CH | 6.48, s | 95.0, CH |
| 9 | | 152.2, C | | 153.7, C[c] | | 152.1, C | | 153.8, C[c] |
| 10 | | 106.1, C | | 106.3, C | | 106.1, C | | 106.3, C |
| 1' | | 122.1, C | | 122.9, C | | 122.0, C | | 122.4, C |
| 2' | 7.66, d (1.7) | 128.2, CH | 7.63, d (1.5) | 129.3, CH | 7.60, d (2.1) | 124.6, CH | 7.88, d (2.2) | 133.1, CH |
| 3' | | 118.6, C | | 121.3, C | | 120.7, C | | 127.3, C |
| 4' | | 153.2, C | | 155.0, C | | 153.2, C | | 160.1, C |
| 5' | | 130.3, C | | 132.1, C | | 130.2, C | 6.88, d (8.5) | 116.3, CH |
| 6' | 7.76, d (1.7) | 128.0, CH | 7.68, d (1.5) | 129.1, CH | 7.75, d (2.1) | 130.0, CH | 7.82, dd (8.5, 2.2) | 129.5, CH |
| 1'' | 3.16, dd (16.7, 4.8)<br>2.87, dd (16.7, 5.5) | 31.5, $CH_2$ | 3.07, dd (16.6, 5.2)<br>2.78, dd (16.6, 7.5) | 32.3, $CH_2$ | 6.39, d (9.8) | 122.1, CH | 2.96, dd (13.7, 5.2)<br>2.83, dd (13.7, 7.8) | 38.1, $CH_2$ |
| 2'' | 3.88, dd (5.5, 4.8) | 69.4, CH | 3.80, dd (7.5, 5.2) | 70.1, CH | 5.69, d (9.8) | 130.8, CH | 4.39, dd (7.8, 5.2) | 76.4, CH |
| 3'' | | 77.7, C | | 79.1, C | | 77.2, C | | 148.6, C |
| 4'' | 1.41, s | 22.4, $CH_3$ | 1.30, s | 21.3, $CH_3$ | 1.48, s | 28.3, $CH_3$ | 4.77, s; 4.87, s | 111.5, $CH_2$ |
| 5'' | 1.36, s | 25.1, $CH_3$ | 1.39, s | 26.1, $CH_3$ | 1.48, s | 28.3, $CH_3$ | 1.81, s | 18.1, $CH_3$ |
| 1''' | 3.36, dd (15.5, 7.5)<br>3.32, dd (15.5, 7.5) | 28.4, $CH_2$ | 2.69, m; 2.58, m | 28.8, $CH_2$ | 2.63, m; 2.72, m | 27.0, $CH_2$ | | |
| 2''' | 5.31, t (7.5) | 121.9, CH | 1.73, m; 1.37, m | 34.6, $CH_2$ | 1.74, m; 1.45, m | 33.1, $CH_2$ | | |
| 3''' | | 133.0, C | 1.60, m | 36.2, CH | 1.71, m | 35.2, CH | | |
| 4''' | 1.74, s | 17.9, $CH_3$ | 3.46, dd (10.6, 5.9)<br>3.37, dd (10.6, 6.6) | 68.4, $CH_2$ | 3.58, dd (10.5, 5.5)<br>3.52, dd (10.5, 6.2) | 68.1, $CH_2$ | | |
| 5''' | 1.76, s | 25.8, $CH_3$ | 0.99, d (6.7) | 17.1, $CH_3$ | 1.03, d (6.5) | 16.5, $CH_3$ | | |
| 3-OMe | 3.83, s | 60.1, $CH_3$ | 3.73, s | 60.5, $CH_3$ | 3.84, s | 60.1, $CH_3$ | 3.75, s | 60.6, $CH_3$ |
| 6-OMe | 4.03, s | 60.9, $CH_3$ | 3.86, s | 60.9, $CH_3$ | 4.03, s | 60.8, $CH_3$ | 3.86, s | 61.0, $CH_3$ |
| 5-OH | 12.94, br. s | | | | 12.95, br. s | | | |

[a]Measured at 600 MHz in $CDCl_3$. [b]Measured at 500 MHz in $CD_3OD$. [c]Interchangeable.

HMBC correlations, from a *meta*-coupled aromatic proton at $\delta_H$ 7.88 (1H, d, $J$ = 2.2 Hz) to the methylene of the isoprenoid group at $\delta_C$ 38.1 and the oxygenated carbon at $\delta_C$ 160.1. Therefore, compound **4** was elucidated as 2-[3-(2-hydroxy-3-methylbut-3-en-1-yl)-4-hydroxyphenyl]-5,7-dihydroxy-3,6-dimethoxy-4*H*-1-benzopyran-4-one and was named dodovisone D.

Compound **5** was isolated as colorless oil. Its molecular formula was determined as $C_{18}H_{26}O_4$ from HREIMS at *m*/*z* 306.1828 $[M]^+$ (calcd 306.1831), requiring six degrees of unsaturation. The IR spectrum showed absorptions at 1773 and 1737, which were attributed to two carbonyl groups. Analysis of the $^{13}C$ NMR data (Table 2) revealed, in addition to a methoxy group at $\delta_C$ 51.6, a total of 17 signals consisting of two methyls, seven methylenes (one oxygenated), three methines (one olefinic), and five quaternary carbons (one olefinic and two carbonylic). The $^1H$ and $^{13}C$ NMR data (Table 2) were in part close to those of mkapwanin,[23] a butenolide-containing clerodane diterpenoid from *D. angustifolia*. However, a methoxy signal and the molecular formula ($C_{18}H_{26}O_4$) required a tri-norclerodane diterpenoid. The absence of signals for butenolide moiety and HMBC correlations (Figure 2) from $H_2$-11, $H_2$-12, and the methoxy protons to C-13 indicated that instead of a butenolide-containing side chain, a methyl propionate one was linked at C-9; therefore, the planar structure of **5** was defined.

The relative configuration of **5** was established by ROESY correlations and proton coupling constants based on computer-generated 3D drawing with minimized energy by MM2 calculation (Figure 2). The *pro*-19*S* diastereotopic proton, which was ω-coupled ($^4J$ = 2.0 Hz) with H-6*β*,[23] showed correlations with H-1*α* and $H_3$-20, indicating *α*- and axial orientation for both C-20 and C-19. In addition, the axially

**Table 2. NMR spectroscopic data[a] for 5 and 6**

| | **5** | | **6** | |
|---|---|---|---|---|
| no. | $\delta_H$ ($J$ in Hz) | $\delta_C$ | $\delta_H$ ($J$ in Hz) | $\delta_C$ |
| 1$\alpha$ | 0.98, qd (12.5, 3.8) | 19.2, $CH_2$ | 1.47–1.63, overlap | 17.36/17.37, $CH_2$[c] |
| 1$\beta$ | 1.59, m | | 1.47–1.63, overlap | |
| 2$\alpha$ | 2.30, dddd (18.1, 7.4, 3.8, 1.9) | 27.4, $CH_2$[b] | 2.13–2.37, overlap | 27.3, $CH_2$ |
| 2$\beta$ | 2.07, dddd (18.1, 11.7, 4.8, 2.1) | | 2.13–2.37, overlap | |
| 3 | 6.66, dd (7.4, 2.1) | 136.2, CH | 6.83, br. s | 140.1, CH |
| 4 | | 138.0, C | | 141.4, C |
| 5 | | 45.3, C | | 37.5, C |
| 6$\alpha$ | 1.79, dt (13.1, 3.2) | 34.1, $CH_2$ | 2.42, br. d (12.0) | 35.6, $CH_2$ |
| 6$\beta$ | 1.13, dddd (13.1, 13.1, 3.7, 2.0) | | 1.15, br. t (11.5) | |
| 7$\alpha$ | 1.39, m | 27.3, $CH_2$[b] | 1.38–1.52, overlap | 27.1, $CH_2$ |
| 7$\beta$ | 1.54, m | | 1.38–1.52, overlap | |
| 8 | 1.43, m | 36.3, CH | 1.47, overlap | 36.22/36.27, CH[c] |
| 9 | | 38.2, C | | 38.66/38.67, C[c] |
| 10 | 1.50, d (12.4) | 47.8, CH | 1.29, d (12.0) | 46.6, CH |
| 11 | 1.66, ddd (14.9, 12.2, 5.0) | 32.0, $CH_2$ | 1.51–1.68, overlap | 34.7, $CH_2$ |
| | 1.59, m | | 1.51–1.68, overlap | |
| 12 | 2.19, ddd (15.3, 12.3, 5.0) | 27.7, $CH_2$ | 2.17/2.34, m[c] | 21.22/21.30, $CH_2$[c] |
| | 1.95, ddd (15.3, 12.2, 4.9 ) | | 2.03/2.17, m[c] | |
| 13 | | 174.4, C | | 168.05/168.14, C[c] |
| 14 | | | 5.87, s | 117.64/117.72, CH[c] |
| 15 | | | | 170.65/170.67, C[c] |
| 16 | | | 5.63/5.64, s[b] | 104.31/104.39, CH[c] |
| 17 | 0.75, d (6.4) | 15.1, $CH_3$ | 0.81/0.82, d (5.6)[b] | 15.9, $CH_3$ |
| 18 | | 170.1, C | | 172.1, C |
| 19 | *pro-R* 4.22, d (8.1) | 71.9, $CH_2$ | 1.25, s | 20.4, $CH_3$ |
| | *pro-S* 3.84, dd (8.1, 2.0) | | | |
| 20 | 0.52, s | 17.1, $CH_3$ | 0.79, s | 18.19/18.22, $CH_3$[c] |
| OMe | 3.58, s | 51.6, $CH_3$ | 3.57, s | 57.03/57.16, $CH_3$[c] |

[a]Measured at 600 MHz in $CDCl_3$. [b]Interchangeable. [c]Doubled signals due to epimer (1:1) at C-16.

oriented proton H-6$\beta$ at $\delta_H$ 1.13 (1H, dddd, 13.1, 13.1, 3.7, 2.0) showed correlations with H-8 and H-10, revealing $\beta$- and axial orientation for H-8 and H-10. Thus, compound **5** was finally established as shown and was named dodovislactone A.

Compound **6** was isolated as an inseparable C-16 epimeric mixture (1:1) where some of the signals appeared as duplicate in the NMR spectra (Table 2). HREIMS gave a molecular formula of $C_{21}H_{30}O_5$. Its NMR data indicated a clerodane diterpenoid similar to 16-hydroxycleroda-3,13-dien-16,15-olide-18-oic acid,[24] except that a methoxy group was evident in **6**. HMBC correlation in **6** from the methoxy protons ($\delta_H$ 3.57) to C-16 verified that the methoxy group was at C-16. In the ROESY spectrum, correlations of $H_3$-19/$H_3$-20 and H-10/H-12 revealed the orientation of C-19, C-20, and H-10 as $\alpha$, $\alpha$, and $\beta$, respectively. However, no useful information about the stereochemistry at C-8 was obtained. In consideration of the co-occurrence of **5**, 15-methoxypatagonic acid, 6$\alpha$-hydroxycleroda-3,13-dien-16,15-olid-18-oic acid, and hautriwaic acid, C-8 should have the $R$* configuration of these analogues. This deduction was also supported by referring to the $^1$H and $^{13}$C NMR data of the diterpenoid hardwickiic acid[25] recorded in the same solvent. The diterpenoid was therefore elucidated as shown and was named dodovislactone B.

**Figure 2.** Key HMBC and ROESY correlations of **5**

## Experimental Section

**General Experimental Procedures.** Optical rotations were measured on a Jasco P-1020 automatic digital polarimeter. UV spectra were obtained in an HPLC (Agilent 1200, DAD). IR

spectra were obtained using a Bruker Tensor 27 FT-IR spectrometer with KBr pellets. The NMR spectra were acquired with an Avance III 600 or Bruker DRX-500 instrument at room temperature. EIMS (including HREIMS) were measured on a VG-Auto-Spec-3000 spectrometer. ESIMS were measured on API QSTAR Pulsar i mass spectrometers. Silica gel (200–300 mesh, Qingdao Marine Chemical Inc., China) and Sephadex LH-20 (Amersham Biosciences, Sweden) were used for column chromatography. Fractions were monitored by TLC (Qingdao Marine Chemical Inc., China) in combination with reversed-phase HPLC (Agilent 1200, Extend-C18 column, 5 $\mu$m, 4.6 × 150 mm). Prep. HPLC was performed using an Agilent 1100 series (ZORBAX SB-C18 column, 5 $\mu$m, 9.4 × 150 mm for 10 mL/min). Silica gel for prep. TLC was obtained from Qingdao Marine Chemical Inc., China.

**Plant Material.** The aerial parts of *D. viscosa* were collected from Yuanyang County in Yunnan Province, China, on May 2011 and were identified by Prof. Yu Chen of Kunming Institute of Botany, Chinese Academy of Sciences. The voucher specimen was deposited at BioBioPha Co., Ltd.

**Extraction and Isolation.** The aerial parts of *D. viscosa* (12 kg) was powdered and extracted with MeOH at room temperature. After filtration, the methanolic extract was evaporated under reduced pressure to get a residue (*ca.* 900 g), which was fractionized by silica gel column chromatography using petroleum ether (PE)/acetone gradient and then MeOH to yield six main fractions A–F. Separation of fraction C eluted with PE/acetone (9:1→7:3) by silica gel eluted with $CHCl_3$/MeOH gave four subfractions C1 (100:1), C2 (30:1), C3 (20:1), and C4 (10:1). Fr. C1 was purified by Sephadex LH-20 ($CHCl_3$/MeOH, 1:1) and then preparative TLC (PE/EtOAc, 9:1) to afford **5** (4 mg). Fraction C2 was purified by Sephadex LH-20 ($CHCl_3$/MeOH, 1:1) and then preparative HPLC on a ZORBAX SB-C18 column (20%→100% $CH_3CN$ in $H_2O$ over 10 min) to afford **3** (4 mg, $t_R$ = 7.1 min) and **1** (2 mg, $t_R$ = 7.6 min). Fraction C3 was purified by silica gel ($CHCl_3$/MeOH, 20:1) and then Sephadex LH-20 (MeOH) to afford **2** (62 mg) and **4** (2 mg). Fraction D eluted by PE/acetone (6:4→1:1) was further separated by silica gel ($CHCl_3$/MeOH, 50:1) into two subfractions - D1 and D2. After repeated preparative TLC (PE/EtOAc/formic acid, 40:10:0.2), fraction D1 afforded **6** (23 mg).

**Dodovisone A (1):** yellowish powder; $[\alpha]_D^{18}$ – 18.7 (*c* 0.13, $CHCl_3$); UV (MeOH) $\lambda_{max}$: 251, 271, 352 nm; IR (KBr) $\nu_{max}$ 3417, 2975, 2925, 2852, 1656, 1612, 1591, 1561, 1468, 1368, 1308, 1209, 1170, 1139, 1093, 1049, 952 $cm^{-1}$; $^1$H and $^{13}$C NMR data (see Table 1); EIMS: *m/z* 482 $[M]^+$ (100), 467 (25), 439 (10), 413 (12), 395 (11), 355 (12), 337 (6), 269 (8), 183 (8), 105 (17); HREIMS: *m/z* 482.1960 $[M]^+$ (calcd for $C_{27}H_{30}O_8$, 482.1941).

**Dodovisone B (2):** yellowish powder; $[\alpha]_D^{19}$ – 3.8 (*c* 0.21, MeOH); UV (MeOH) $\lambda_{max}$: 212, 251, 271, 353 nm; IR (KBr) $\nu_{max}$ 3425, 2972, 2932, 2873, 1654, 1612, 1594, 1562, 1468, 1369, 1307, 1266, 1210, 1171, 1139, 1092, 1048, 952 $cm^{-1}$; $^1$H and $^{13}$C NMR data (see Table 1); ESIMS (pos.): *m/z* 523 $[M + Na]^+$; HREIMS: *m/z* 500.2034 $[M]^+$ (calcd for $C_{27}H_{32}O_9$, 500.2046).

**Dodovisone C (3):** yellowish powder; $[\alpha]_D^{18}$ – 53.3 (*c* 0.10, $CHCl_3$); UV (MeOH) $\lambda_{max}$: 216, 243, 272, 357 nm; IR (KBr) $\nu_{max}$ 3416, 2960, 2927, 2854, 1654, 1611, 1562, 1467, 1378, 1365, 1307, 1269, 1209, 1170, 1122, 1092, 1050, 954 $cm^{-1}$; $^1$H and $^{13}$C NMR data (see Table 1); EIMS: *m/z* 482 $[M]^+$ (71), 467 (100), 449 (7), 409 (8), 395 (8), 234 (7), 197 (8), 175 (8), 169 (5), 118 (4); HREIMS: *m/z* 482.1941 $[M]^+$ (calcd for $C_{27}H_{30}O_8$, 482.1941).

**Dodovisone D (4):** yellowish powder; $[\alpha]_D^{19}$ – 9.2 (*c* 0.20, MeOH); UV (MeOH) $\lambda_{max}$: 248, 270, 349 nm; IR (KBr) $\nu_{max}$ 3423, 2933, 2851, 1654, 1610, 1563, 1470, 1366, 1280, 1208, 1169, 1122, 1092, 1046, 997, 807 $cm^{-1}$; $^1$H and $^{13}$C NMR data (see Table 1); EIMS: *m/z* 414 $[M]^+$ (85), 399 (10), 381 (17), 344 (70), 329 (33), 285 (10), 84 (77), 66 (100); HREIMS: *m/z* 414.1345 $[M]^+$ (calcd for $C_{22}H_{22}O_8$, 414.1315).

**Dodovislactone A (5):** colorless oil; $[\alpha]_D^{18}$ – 96.3 (*c* 0.28, $CHCl_3$); UV (MeOH) $\lambda_{max}$: 240 (sh) nm; IR (KBr) $\nu_{max}$ 2957, 2927, 2874, 1773, 1737, 1452, 1437, 1286, 1198, 1189, 1122, 1038, 1005, 988 $cm^{-1}$; $^1$H and $^{13}$C NMR data (see Table 2); ESIMS (pos.): *m/z* 329 $[M + Na]^+$; HREIMS: *m/z* 306.1828 $[M]^+$ (calcd for $C_{18}H_{26}O_4$, 306.1831).

**Dodovislactone B (6):** colorless oil; $[\alpha]_D^{19}$ – 72.8 (*c* 0.20, $CHCl_3$); UV (MeOH) $\lambda_{max}$: 212 nm; IR (KBr) $\nu_{max}$ 3440, 2960, 2923, 2862, 1796, 1765, 1711, 1680, 1648, 1629, 1457, 1420, 1382, 1263, 1204, 1172, 1118, 962, 939 $cm^{-1}$; $^1$H and $^{13}$C NMR data (see Table 2); ESIMS (pos.): *m/z* 385 $[M + Na]^+$; HREIMS: *m/z* 362.2097 $[M]^+$ (calcd for $C_{21}H_{30}O_5$, 362.2093).

## Electronic Supplementary Material

Supplementary material is available in the online version of this article at http://dx.doi.org/10.1007/s13659-013-0053-4 and is accessible for authorized users.

## Acknowledgments

This work was financially supported by National Basic Research Program of China (973 Program, 2009CB522300), the “West Light” program of Chinese Academy of Sciences, and the “Large-scale Compound Library” project of National Development Reform Commission.

**Open Access** This article is distributed under the terms of the Creative Commons Attribution License which permits any use, distribution, and reproduction in any medium, provided the original author(s) and source are credited.

## Electronic supplementary material

Supplementary material, approximately 1.79 MB.
