# Supplementary material for: Isoprenylated flavonoids and clerodane diterpenoids from Dodonaea viscosa
Source: Nat Prod Bioprospect. 2013 Oct 16;3(5):250–5. doi: 10.1007/s13659-013-0053-4 (PMC4131623; doi:10.1007/s13659-013-0053-4)

Electronic Supplementary Material

Isoprenylated flavonoids and clerodane diterpenoids from *Dodonaea viscosa*

Yuan GAO,[a,b,c] Yin-Dong FANG,[b] Ping HAI,[b] Fei WANG,[a,b] and Ji-Kai LIU[a,]*

[a]State Key Laboratory of Phytochemistry and Plant Resources in West China, Kunming Institute of Botany, Chinese Academy of Sciences, Kunming 650201, China

[b]BioBioPha Co., Ltd., Kunming 650201, China

[c]University of Chinese Academy of Sciences, Beijing 100049, China

Received 10 July 2013; Accepted 3 September 2013

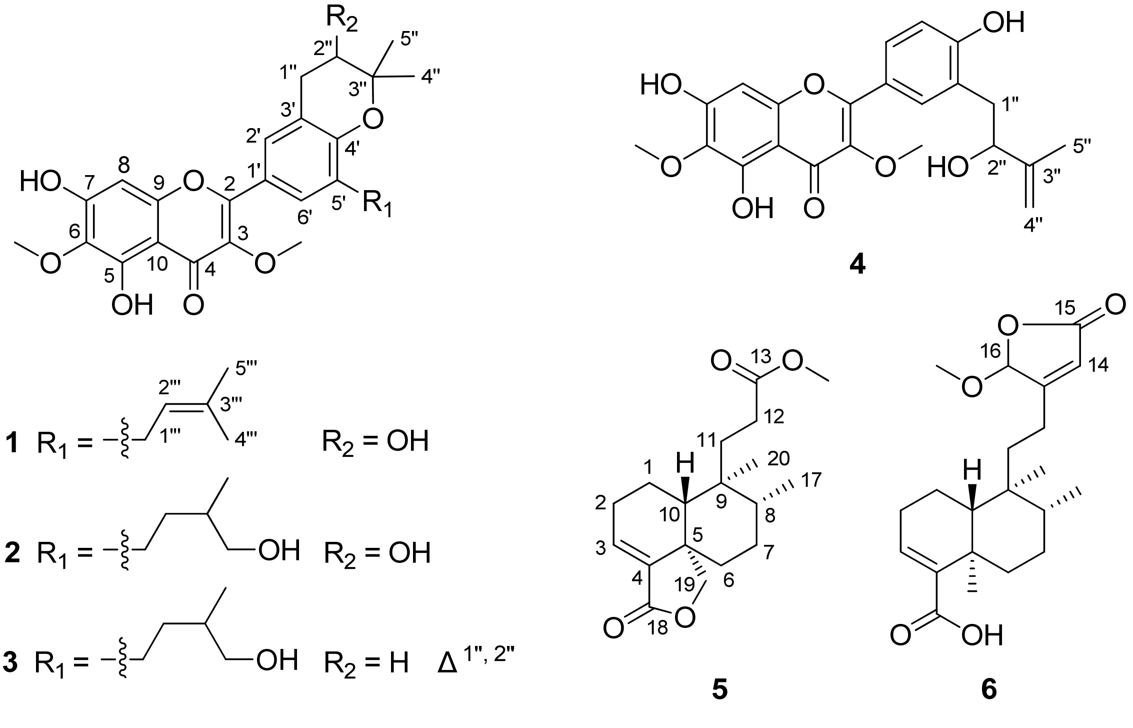

Structures of compounds **1**–**6**

*To whom correspondence should be addressed. E-mail: jkliu@mail.kib.ac.cn

Content list:

S1. ^{1}H NMR spectrum (600 MHz, $CDCl_3$) of dodovisone A (**1**).

S2. ^{13}C NMR (DEPT) spectrum (150 MHz, $CDCl_3$) of dodovisone A (**1**).

S3. HMBC spectrum (600 MHz, $CDCl_3$) of dodovisone A (**1**).

S4. UV spectrum (MeOH) of dodovisone A (**1**).

S5. ^{1}H NMR spectrum (500 MHz, CD_3OD) of dodovisone B (**2**).

S6. ^{13}C NMR (DEPT) spectrum (100 MHz, CD_3OD) of dodovisone B (**2**).

S7. HMBC spectrum (500 MHz, CD_3OD) of dodovisone B (**2**).

S8. UV spectrum (MeOH) of dodovisone B (**2**).

S9. ^{1}H NMR spectrum (600 MHz, $CDCl_3$) of dodovisone C (**3**).

S10. ^{13}C NMR (DEPT) spectrum (150 MHz, $CDCl_3$) of dodovisone C (**3**).

S11. HMBC spectrum (600 MHz, $CDCl_3$) of dodovisone C (**3**).

S12. UV spectrum (MeOH) of dodovisone C (**3**).

S13. ^{1}H NMR spectrum (500 MHz, CD_3OD) of dodovisone C (**4**).

S14. ^{13}C NMR (DEPT) spectrum (100 MHz, CD_3OD) of dodovisone C (**4**).

S15. HMBC spectrum (500 MHz, CD_3OD) of dodovisone C (**4**).

S16. UV spectrum (MeOH) of dodovisone C (**4**).

S17. ^{1}H NMR spectrum (600 MHz, $CDCl_3$) of dodovislactone A (**5**).

S18. ^{13}C NMR spectrum (150 MHz, $CDCl_3$) of dodovislactone A (**5**).

S19. HSQC spectrum (600 MHz, $CDCl_3$) of dodovislactone A (**5**).

S20. HMBC spectrum (600 MHz, $CDCl_3$) of dodovislactone A (**5**).

S21. ROESY spectrum (600 MHz, $CDCl_3$) of dodovislactone A (**5**).

S22. UV spectrum (MeOH) of dodovislactone A (**5**).

S23. ^{1}H NMR spectrum (600 MHz, $CDCl_3$) of dodovislactone B (**6**).

S24. ^{13}C NMR spectrum (150 MHz, $CDCl_3$) of dodovislactone B (**6**).

S25. HSQC spectrum (600 MHz, $CDCl_3$) of dodovislactone B (**6**).

S26. HMBC spectrum (600 MHz, $CDCl_3$) of dodovislactone B (**6**).

S27. ROESY spectrum (600 MHz, $CDCl_3$) of dodovislactone B (**6**).

S28. UV spectrum (MeOH) of dodovislactone B (**6**).

S1. ^{1}H NMR spectrum (600 MHz, $CDCl_3$) of dodovisone A (**1**).

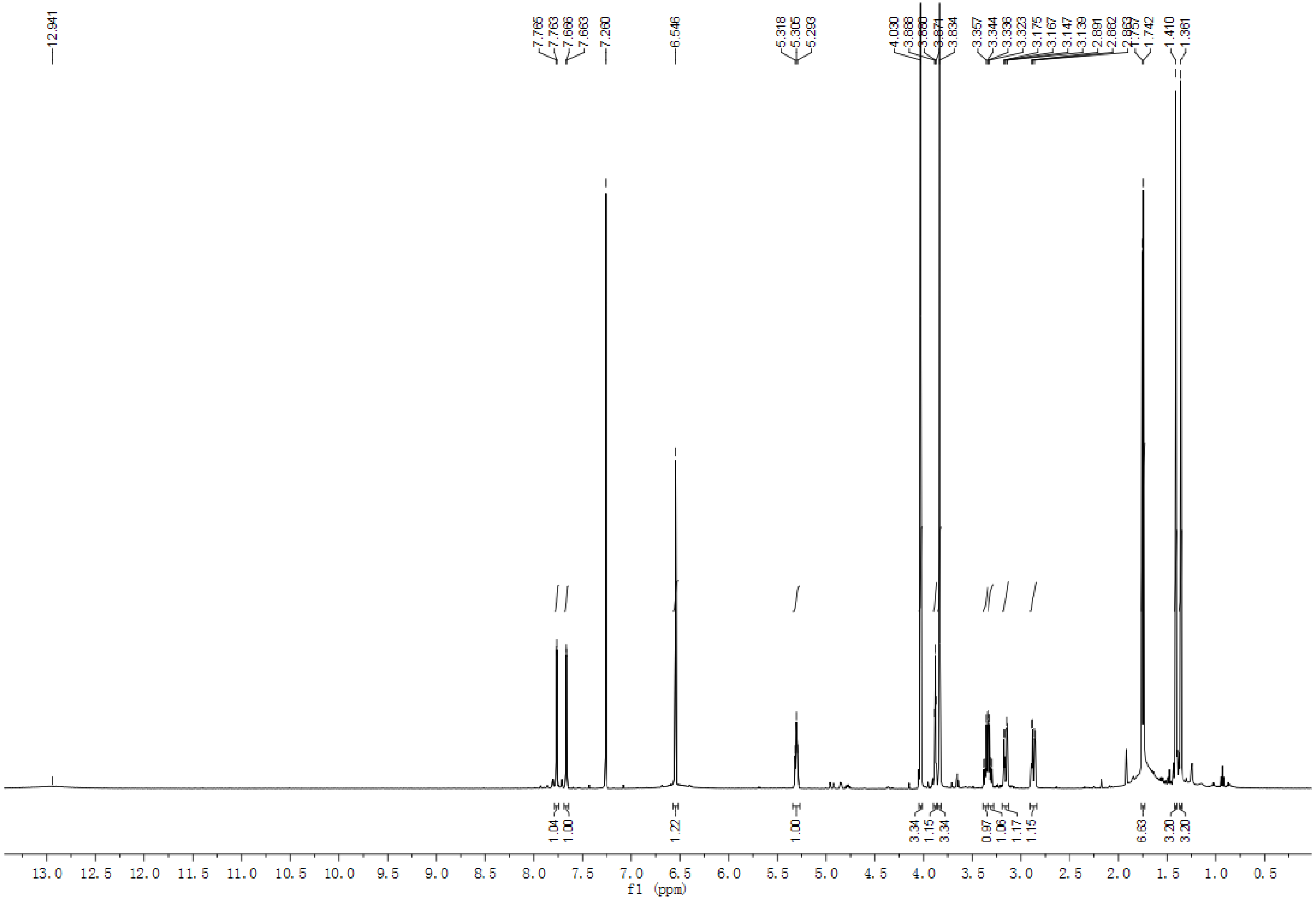

S2. ^{13}C NMR (DEPT) spectrum (150 MHz, $CDCl_3$) of dodovisone A (**1**).

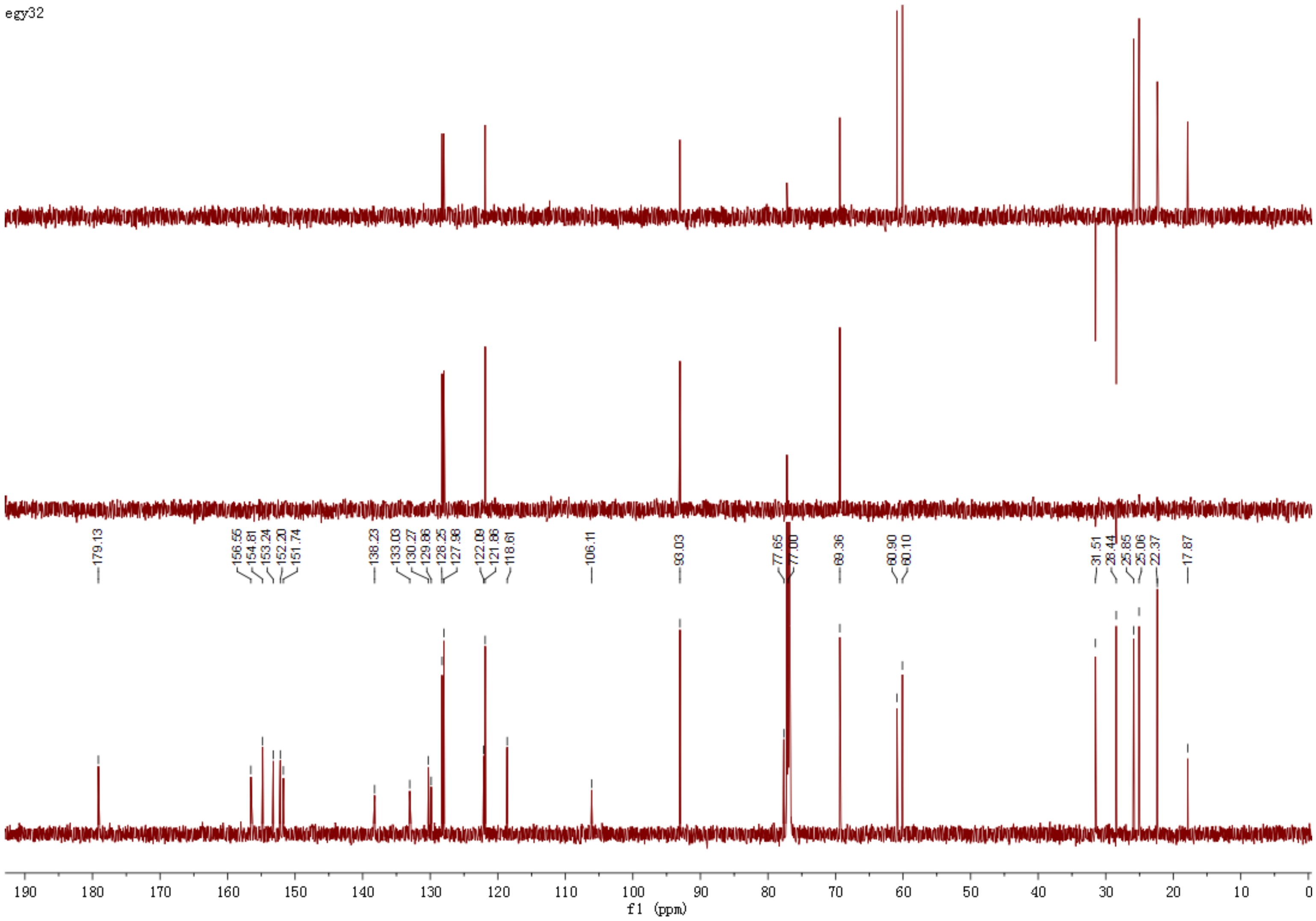

S3. HMBC spectrum (600 MHz, $CDCl_3$) of dodovisone A (**1**).

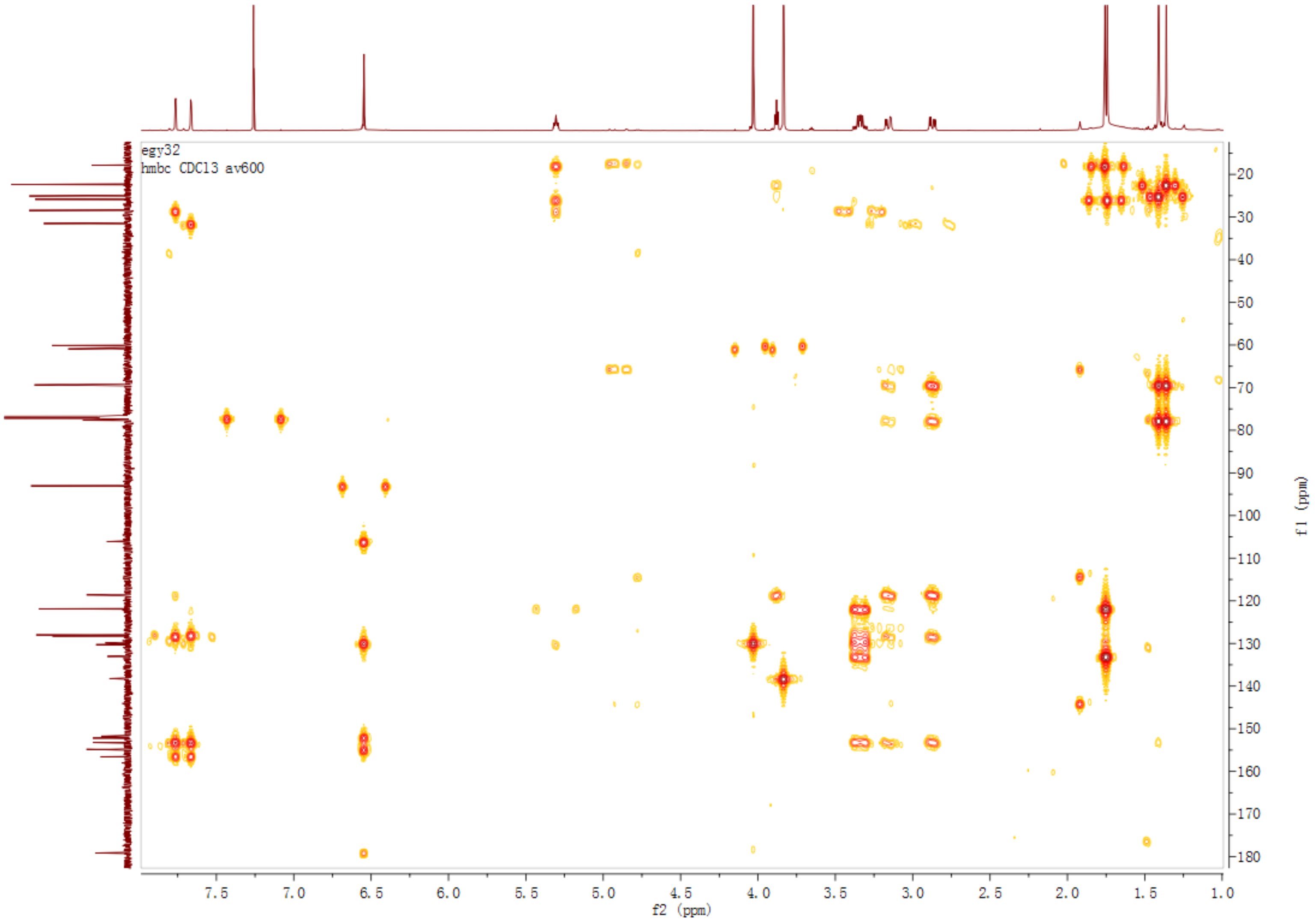

S4. UV spectrum (MeOH) of dodovisone A (**1**).

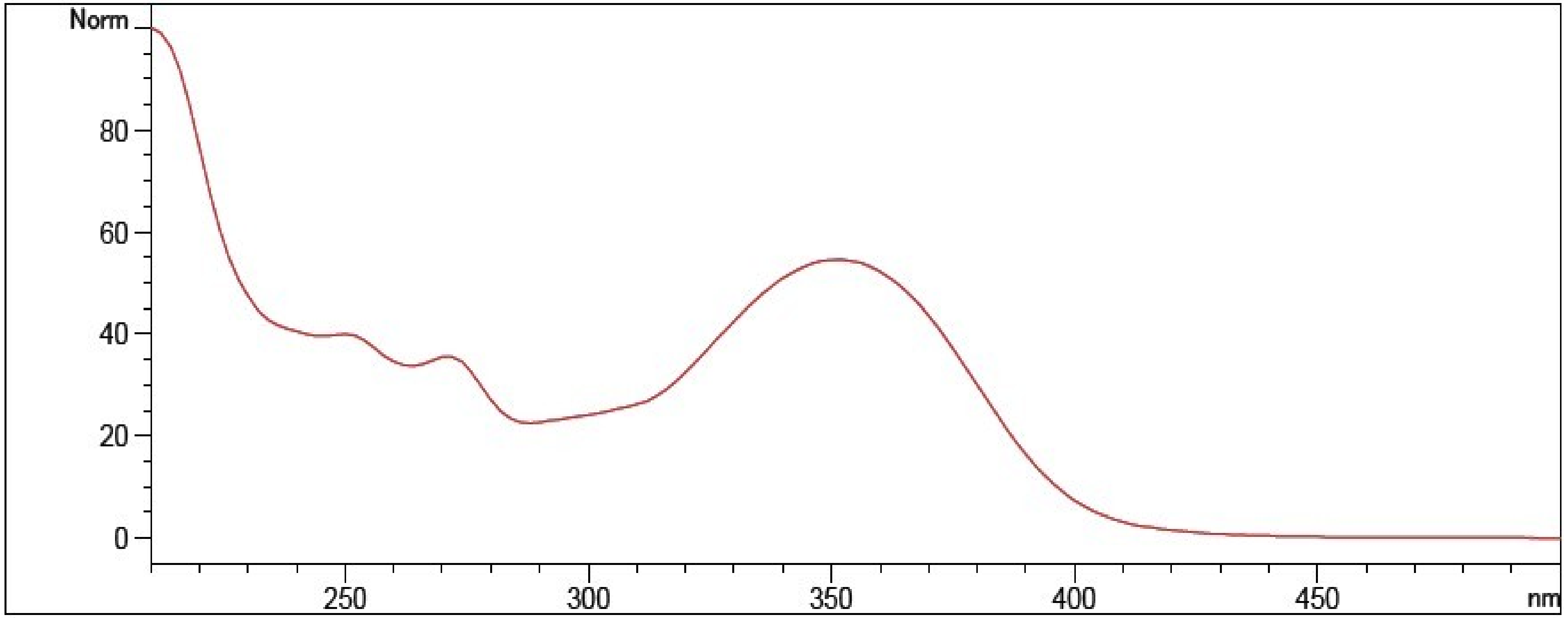

S5. ^{1}H NMR spectrum (500 MHz, CD_3OD) of dodovisone B (**2**).

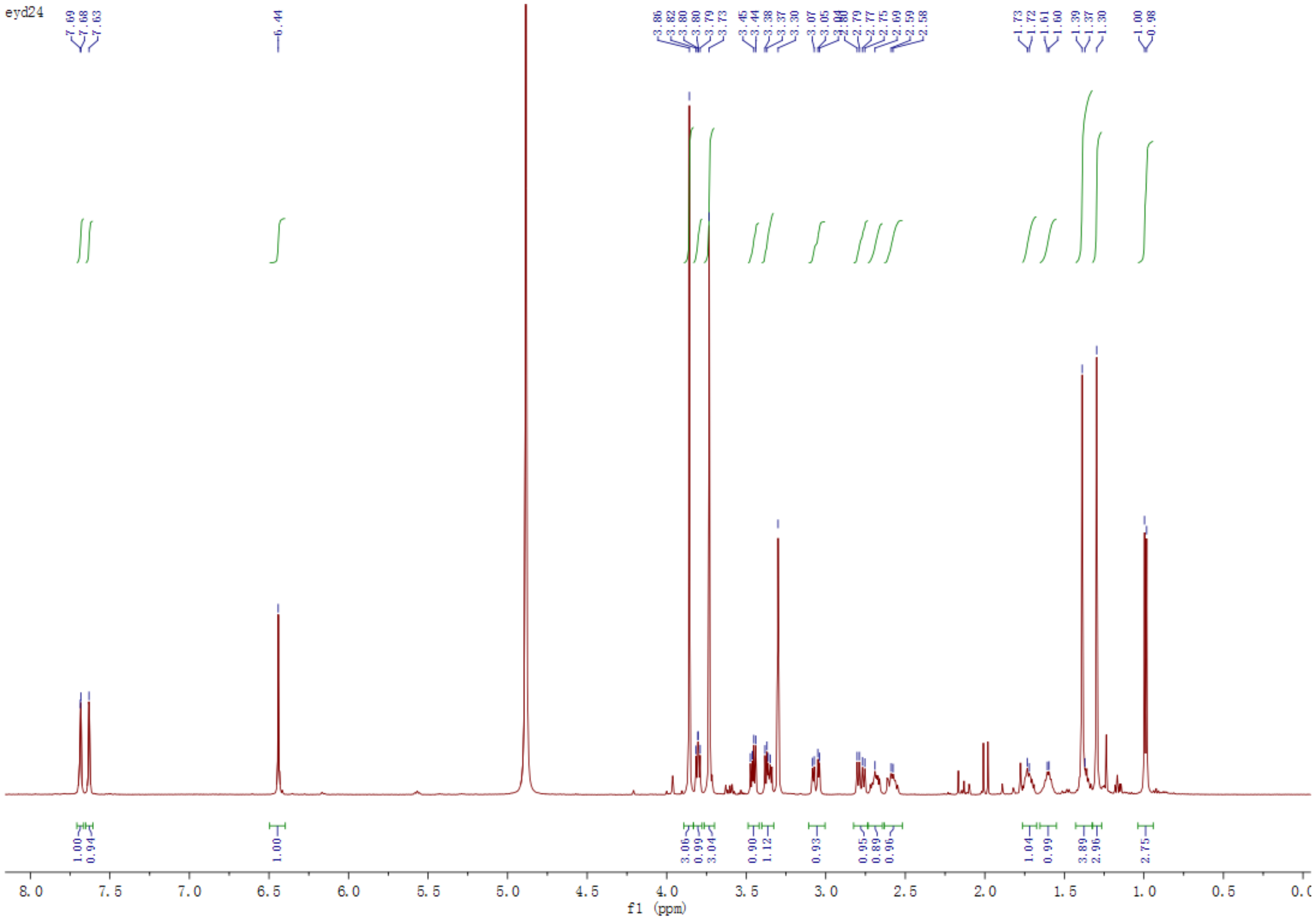

S6. ^{13}C NMR (DEPT) spectrum (100 MHz, CD_3OD) of dodovisone B (**2**).

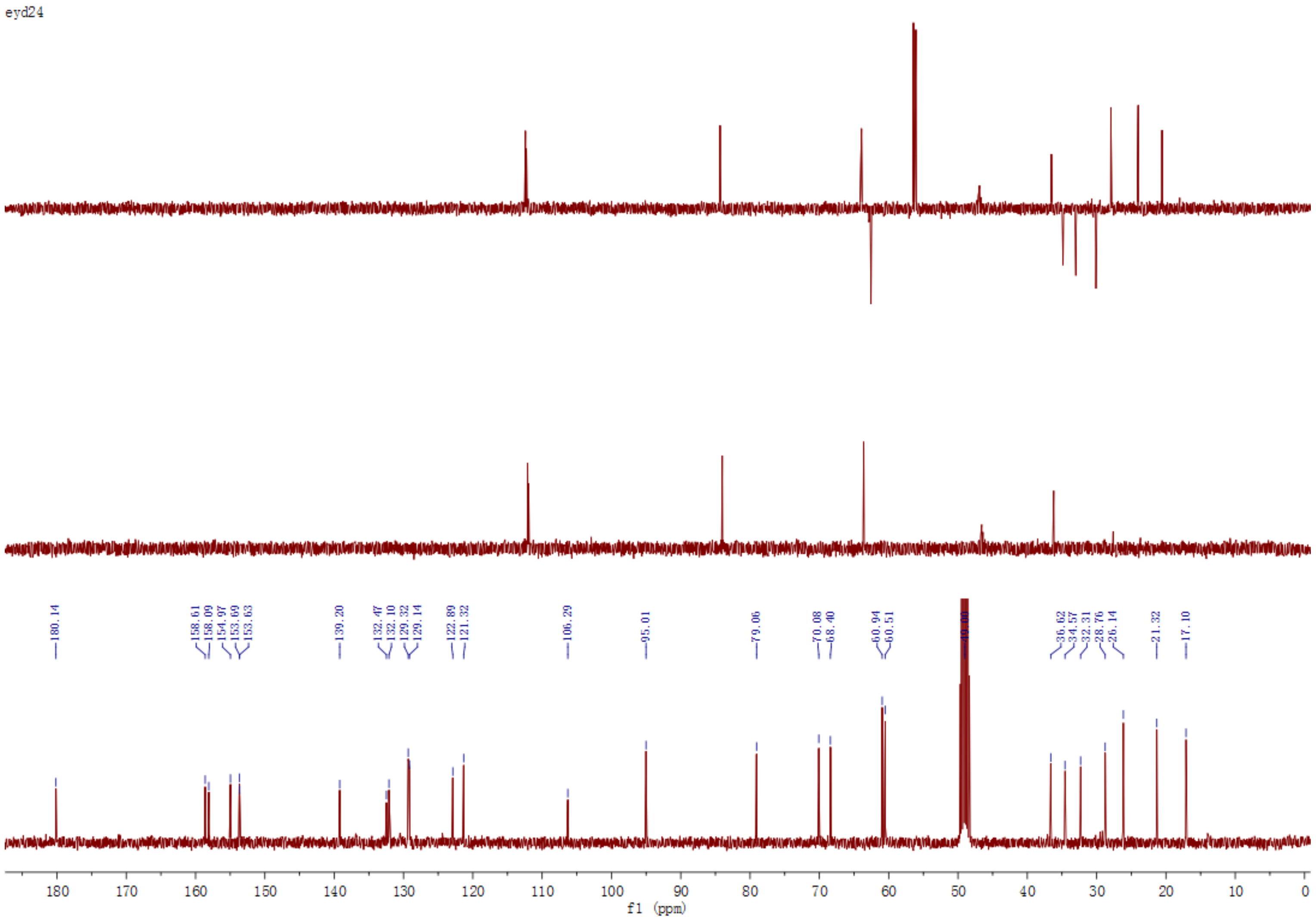

S7. HMBC spectrum (500 MHz, CD_3OD) of dodovisone B (**2**).

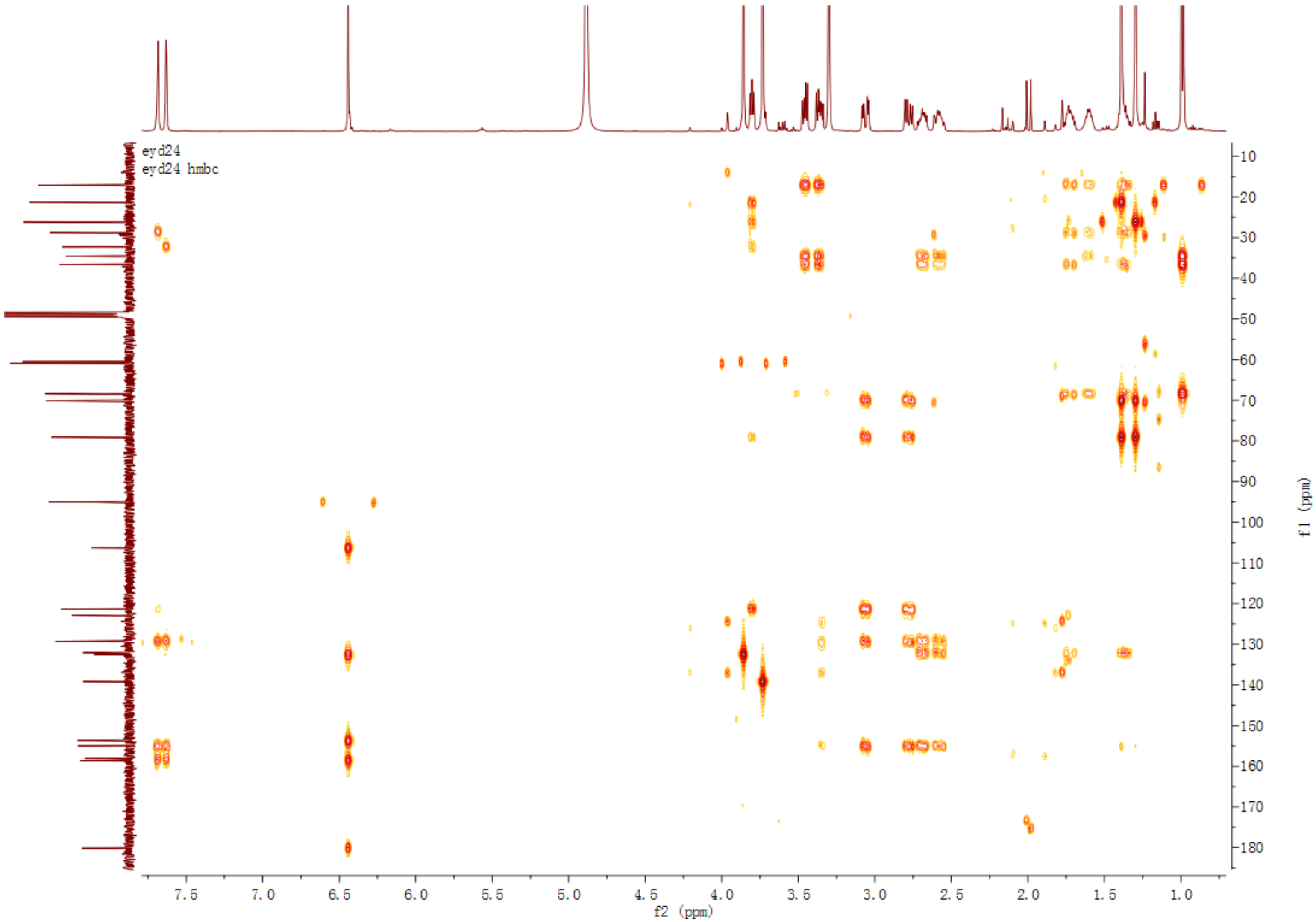

S8. UV spectrum (MeOH) of dodovisone B (**2**).

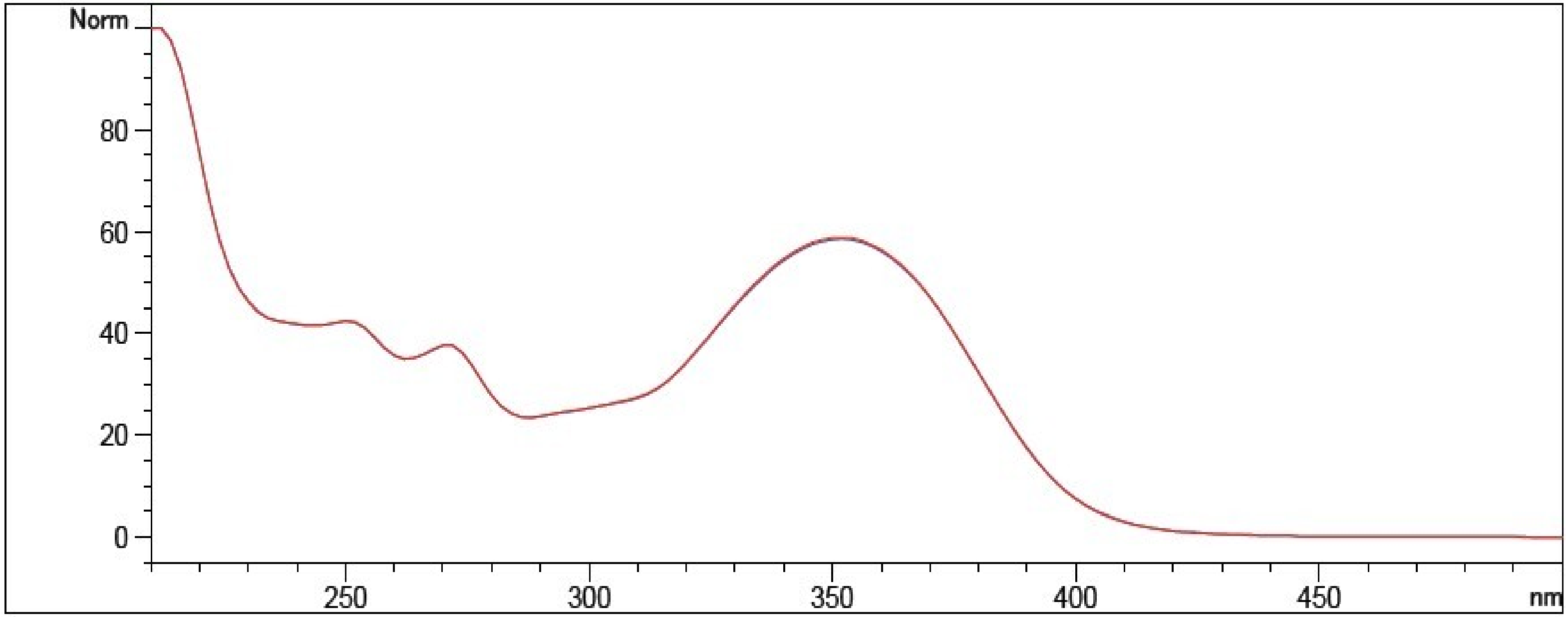

S9. ^{1}H NMR spectrum (600 MHz, $CDCl_3$) of dodovisone C (**3**).

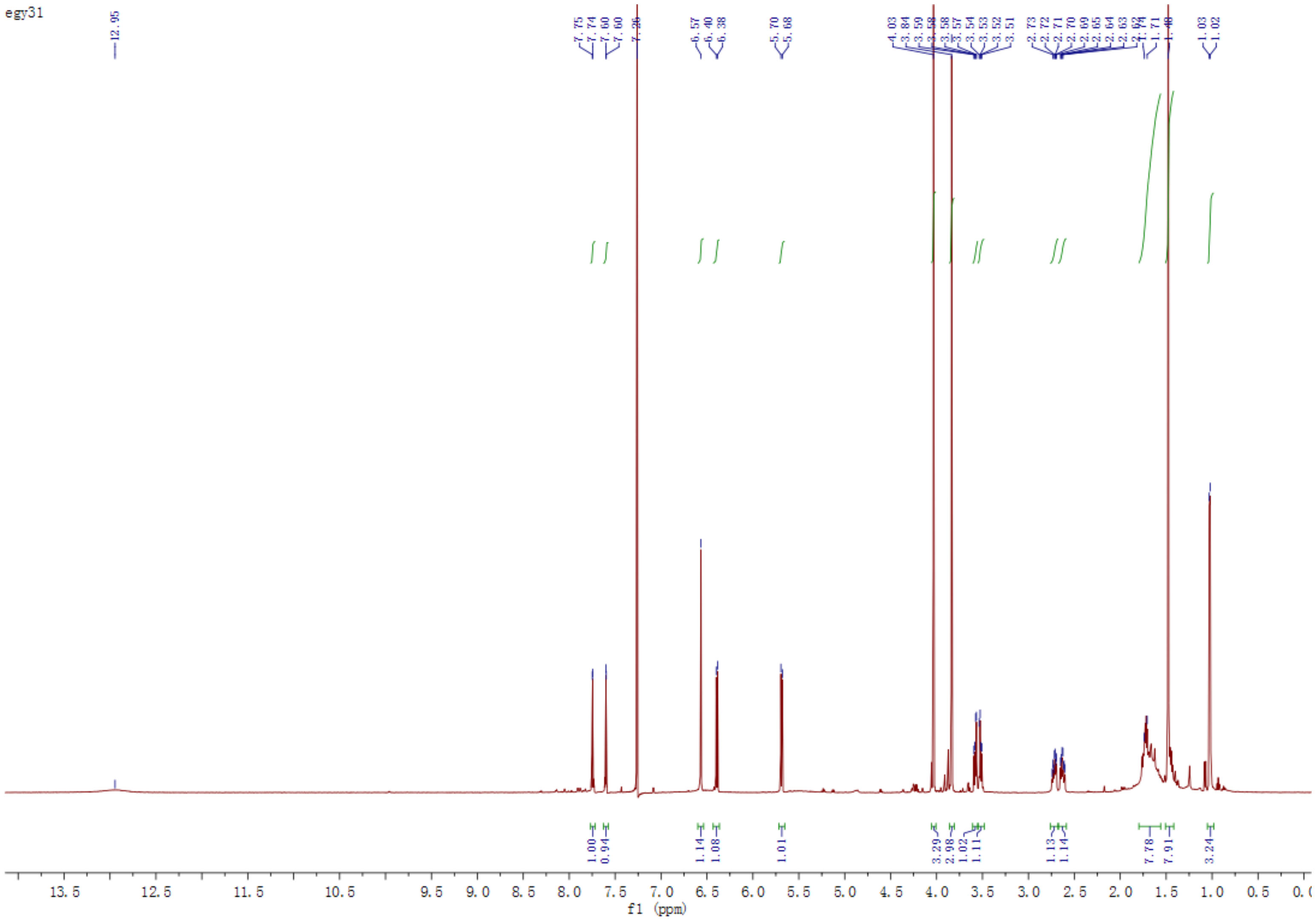

S10. ^{13}C NMR (DEPT) spectrum (150 MHz, $CDCl_3$) of dodovisone C (**3**).

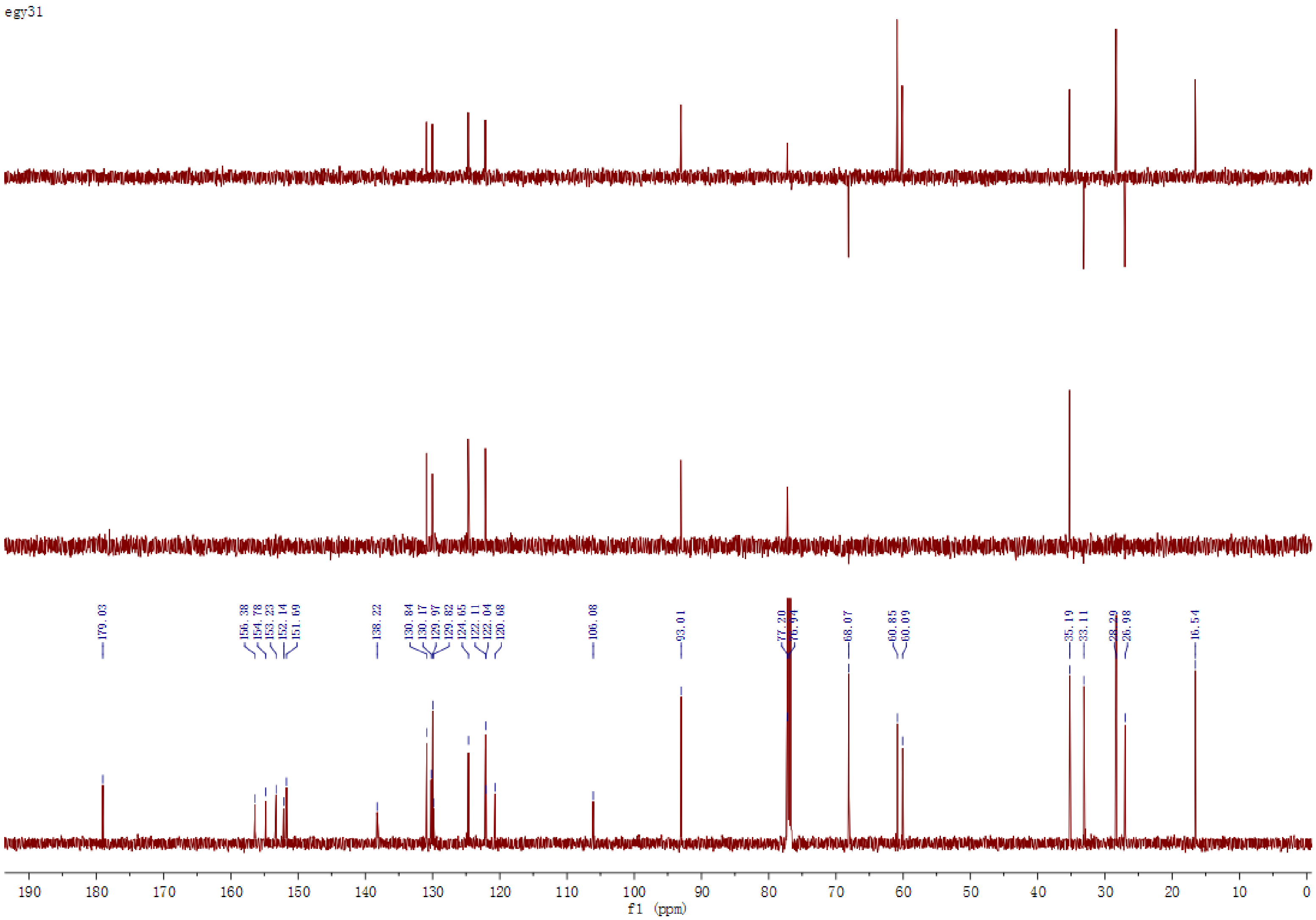

S11. HMBC spectrum (600 MHz, $CDCl_3$) of dodovisone C (**3**).

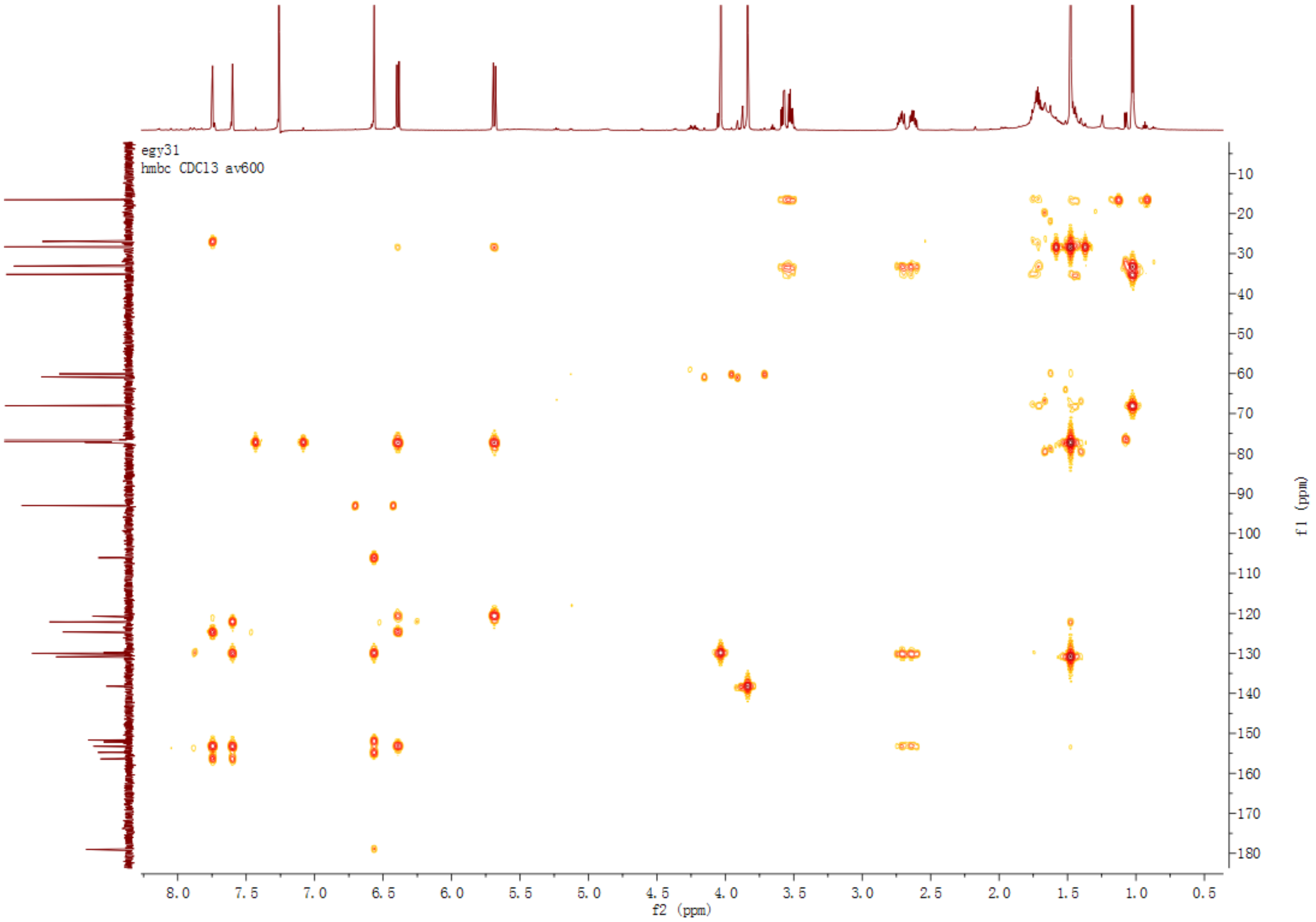

S12. UV spectrum (MeOH) of dodovisone C (**3**).

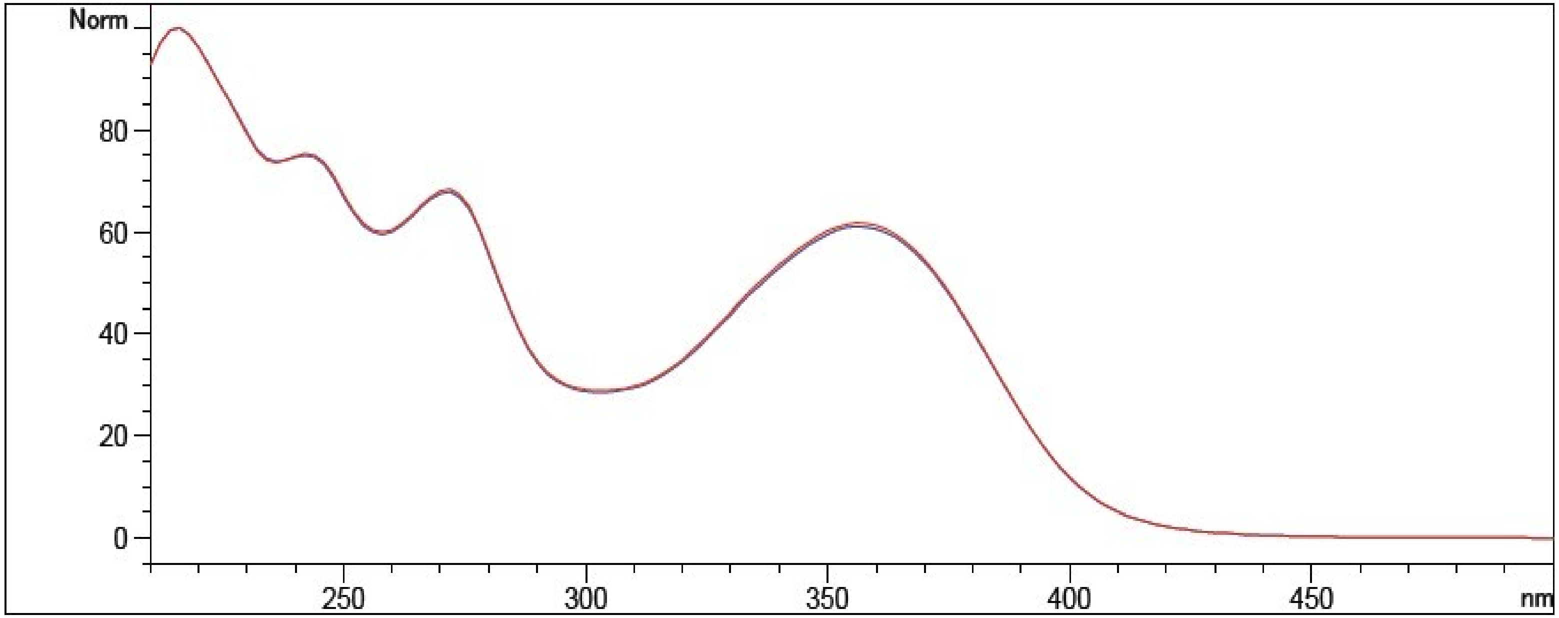

S13. ^{1}H NMR spectrum (500 MHz, CD_3OD) of dodovisone C (**4**).

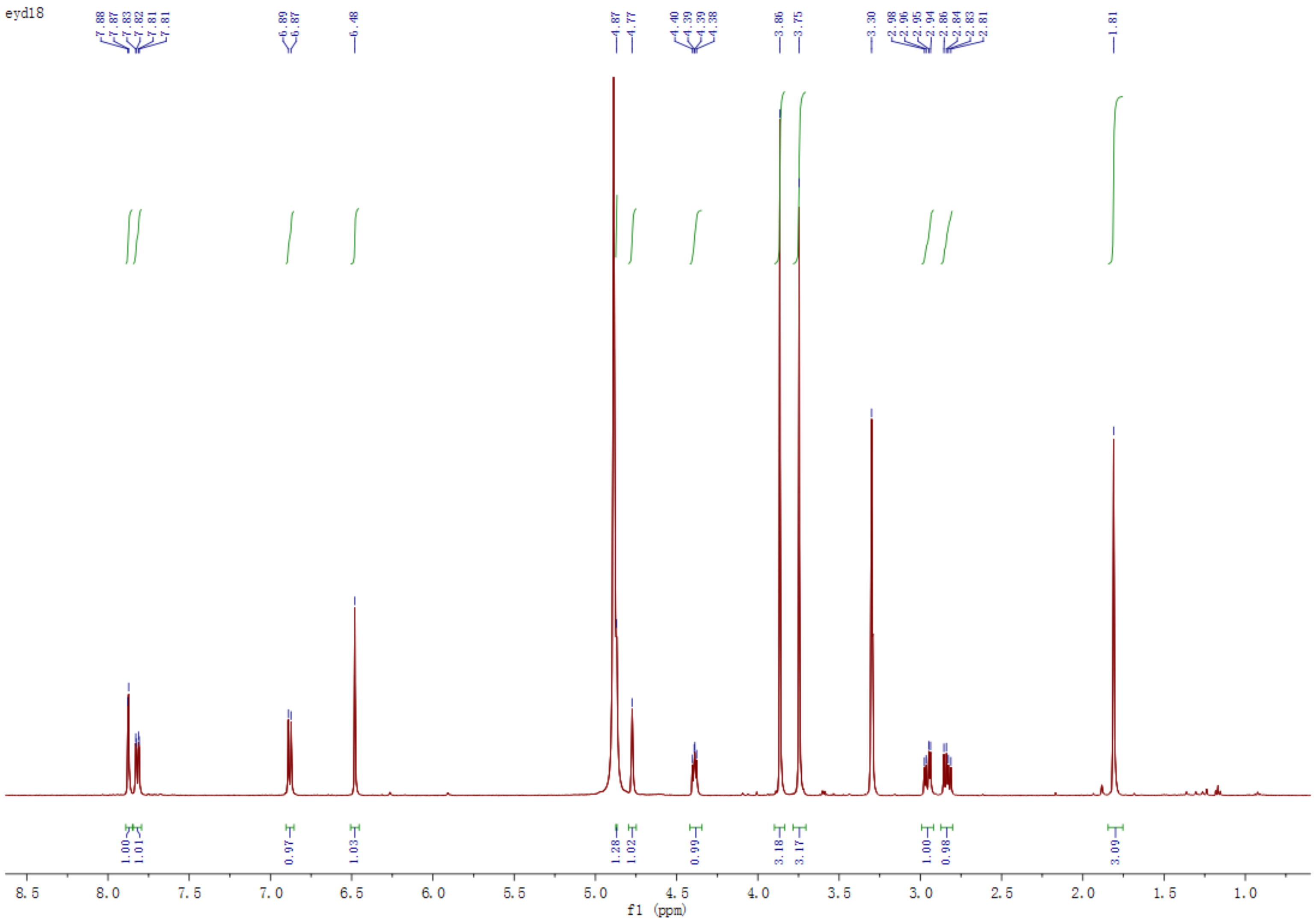

S14. ^{13}C NMR (DEPT) spectrum (100 MHz, CD_3OD) of dodovisone C (**4**).

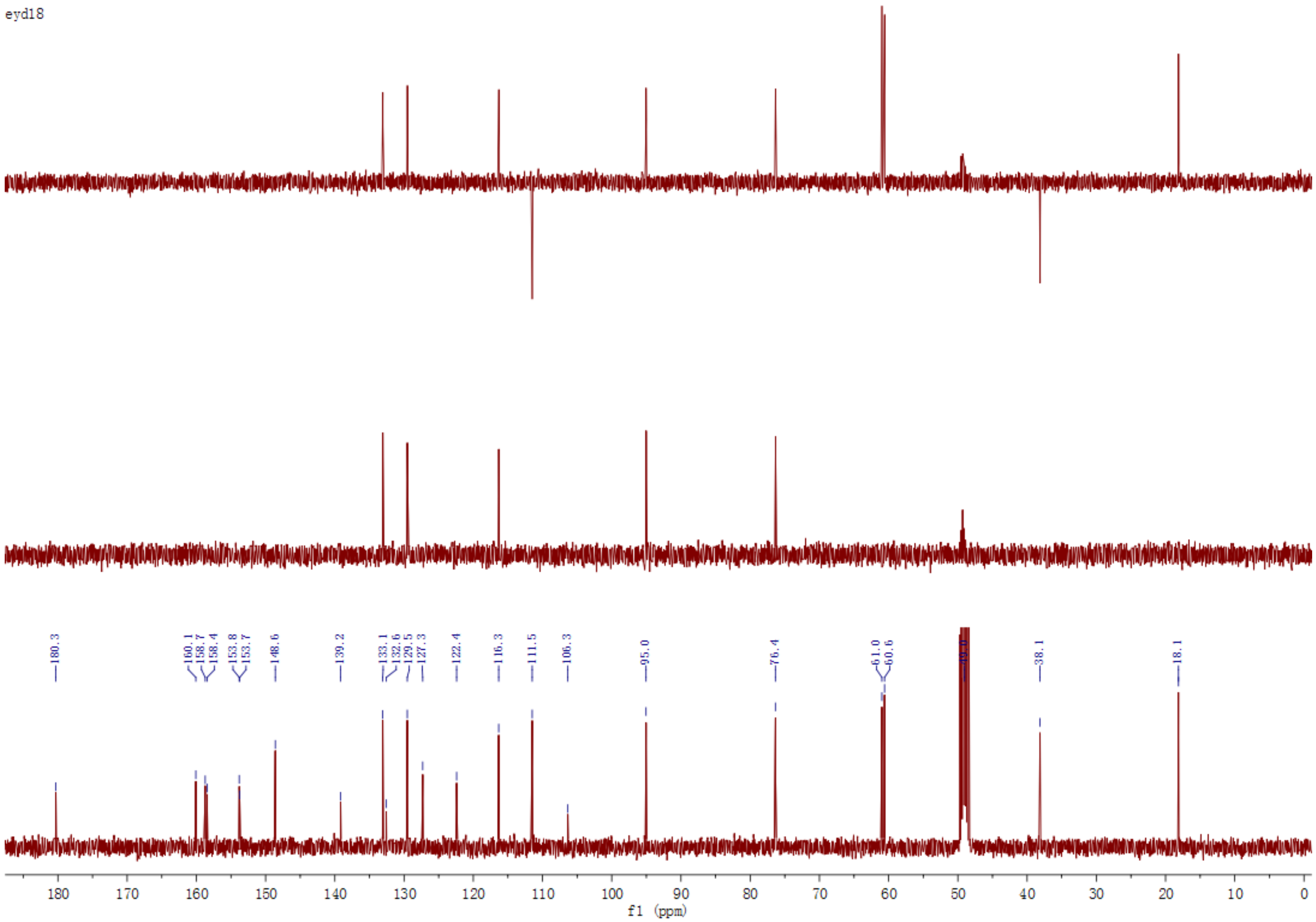

S15. HMBC spectrum (500 MHz, CD_3OD) of dodovisone C (**4**).

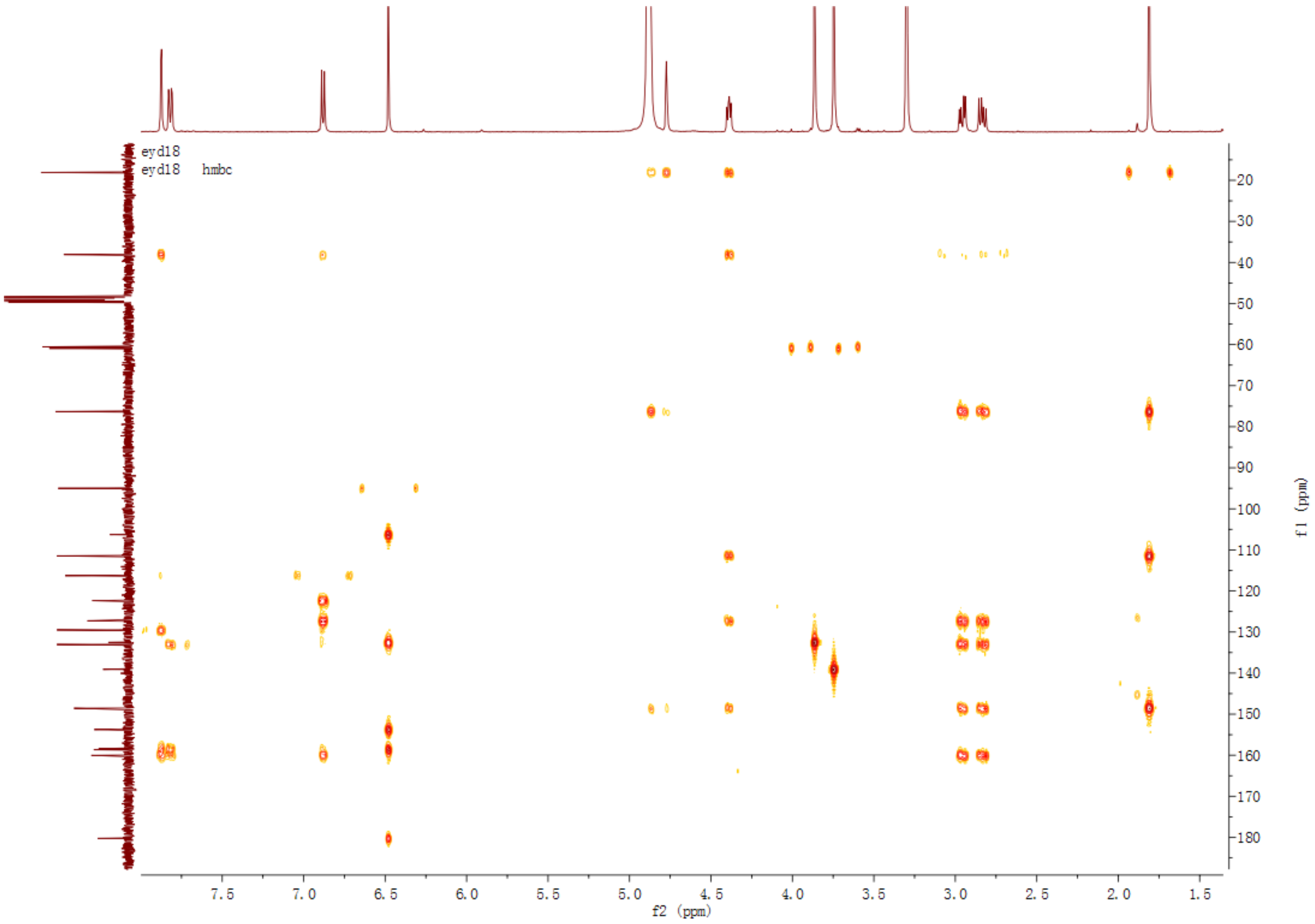

S16. UV spectrum (MeOH) of dodovisone C (**4**).

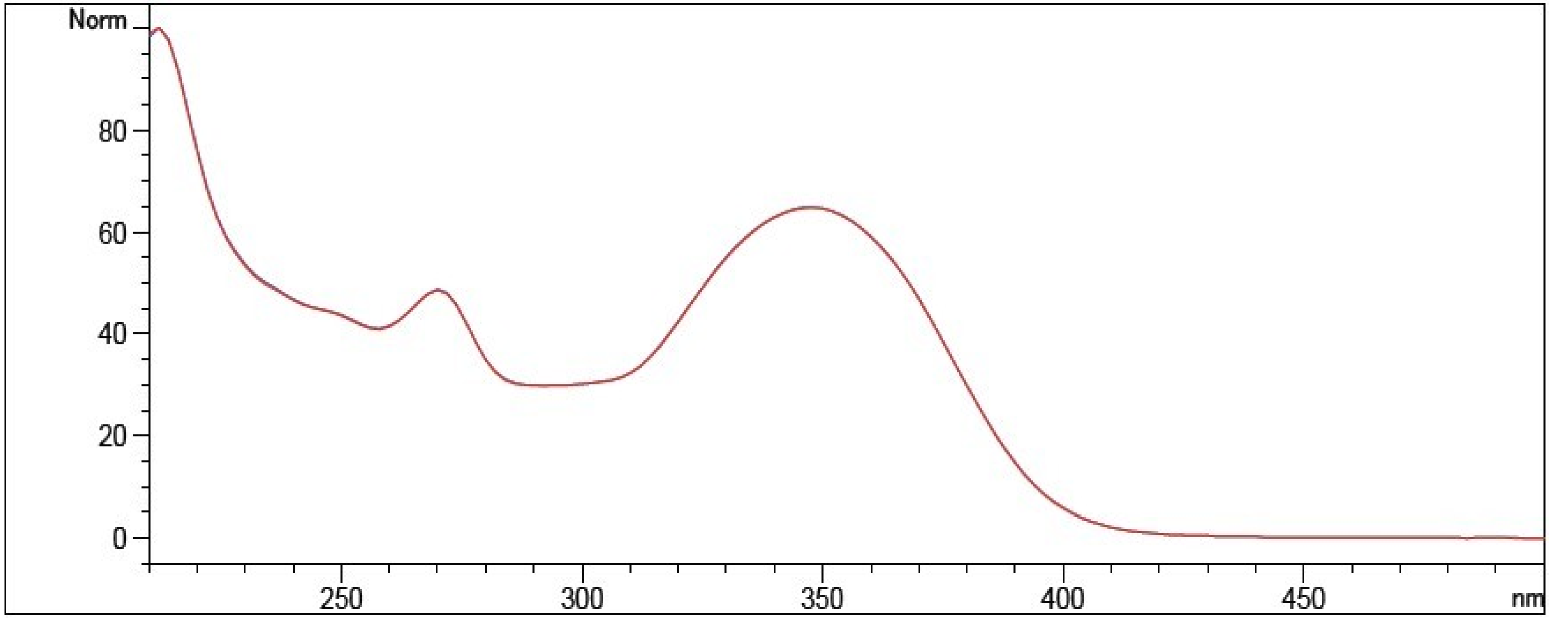

S17. ^{1}H NMR spectrum (600 MHz, $CDCl_3$) of dodovislactone A (**5**).

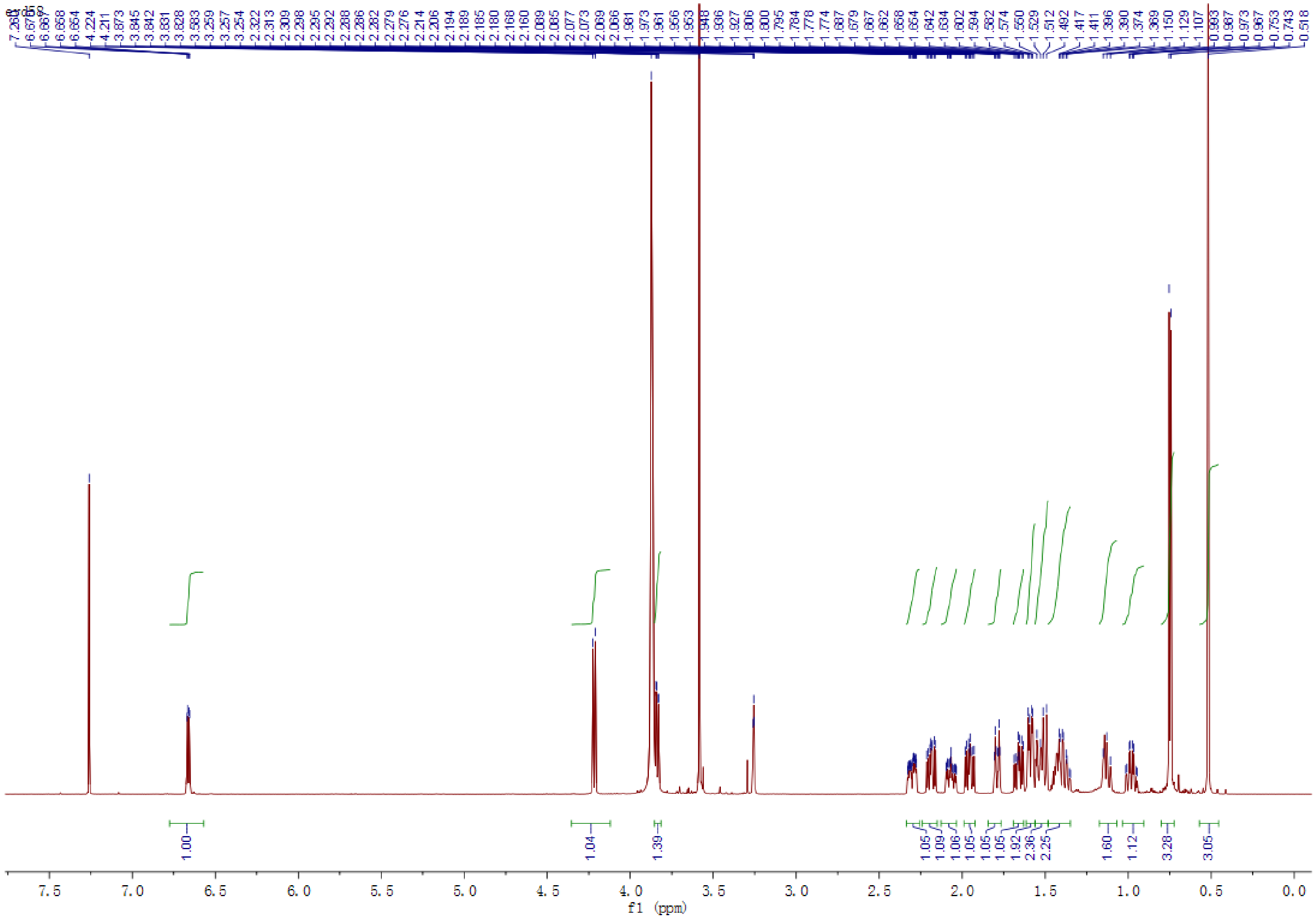

S18. ^{13}C NMR (DEPT) spectrum (150 MHz, $CDCl_3$) of dodovislactone A (**5**).

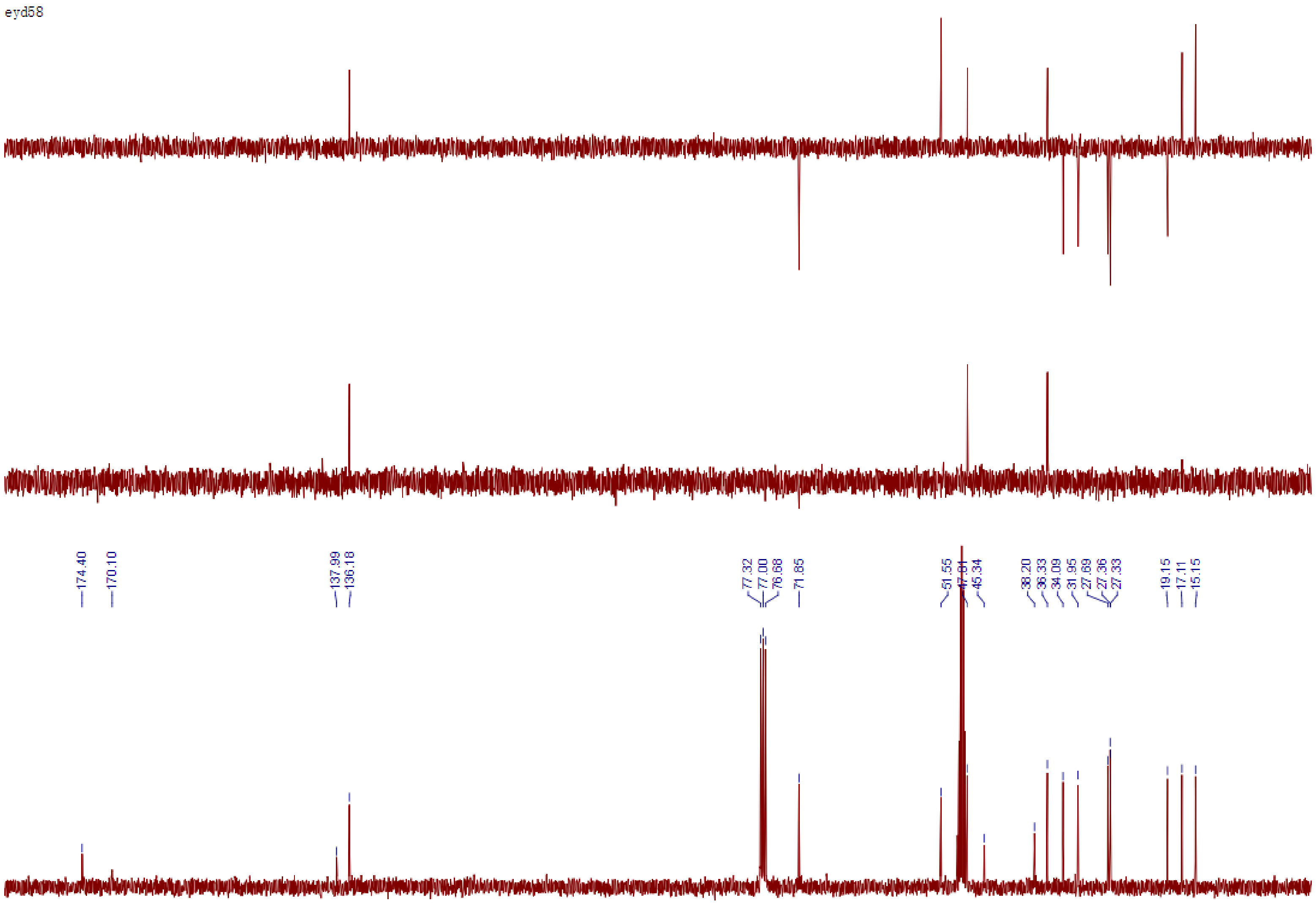

S19. HSQC spectrum (600 MHz, $CDCl_3$) of dodovislactone A (**5**).

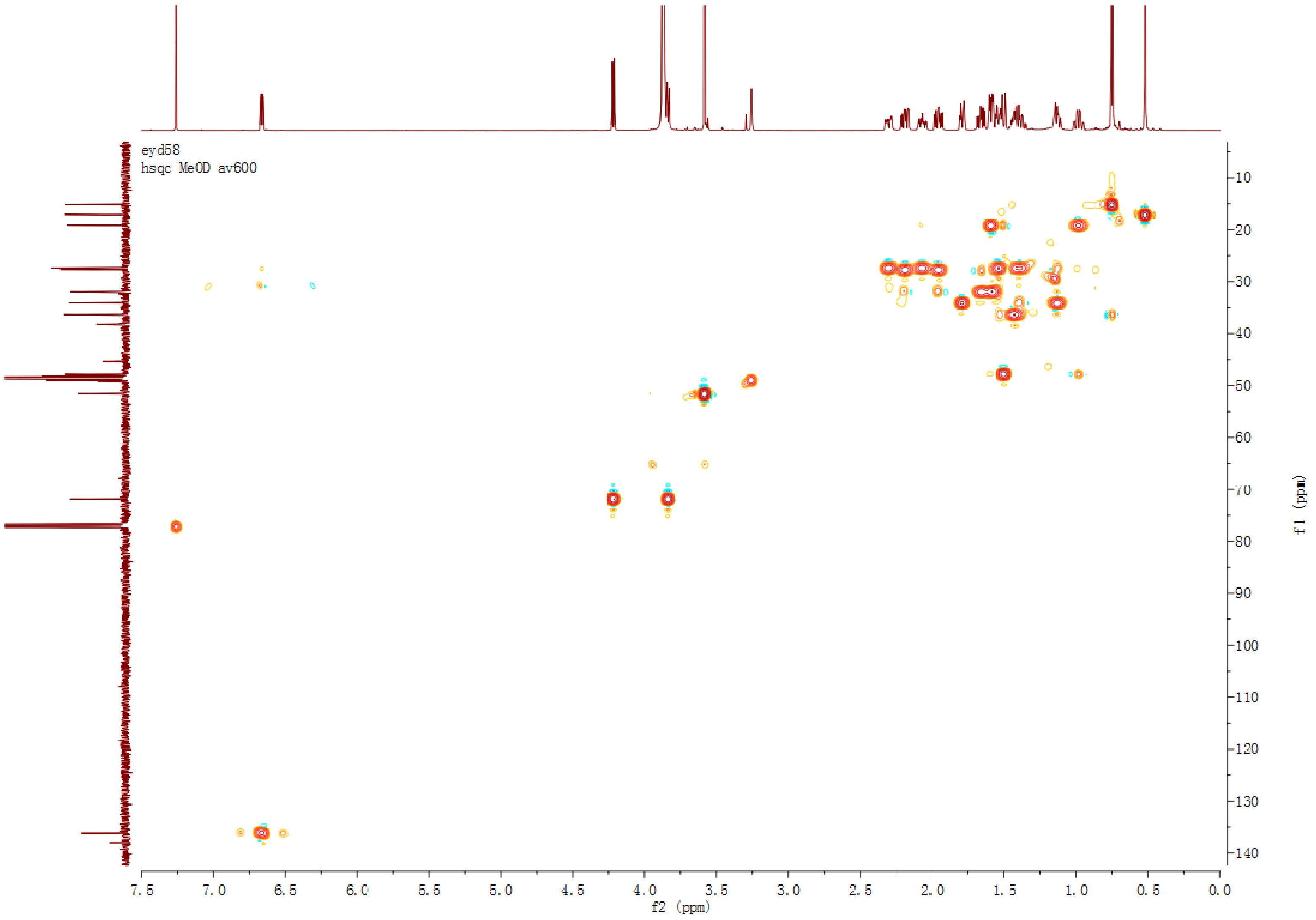

S20. HMBC spectrum (600 MHz, $CDCl_3$) of dodovislactone A (**5**).

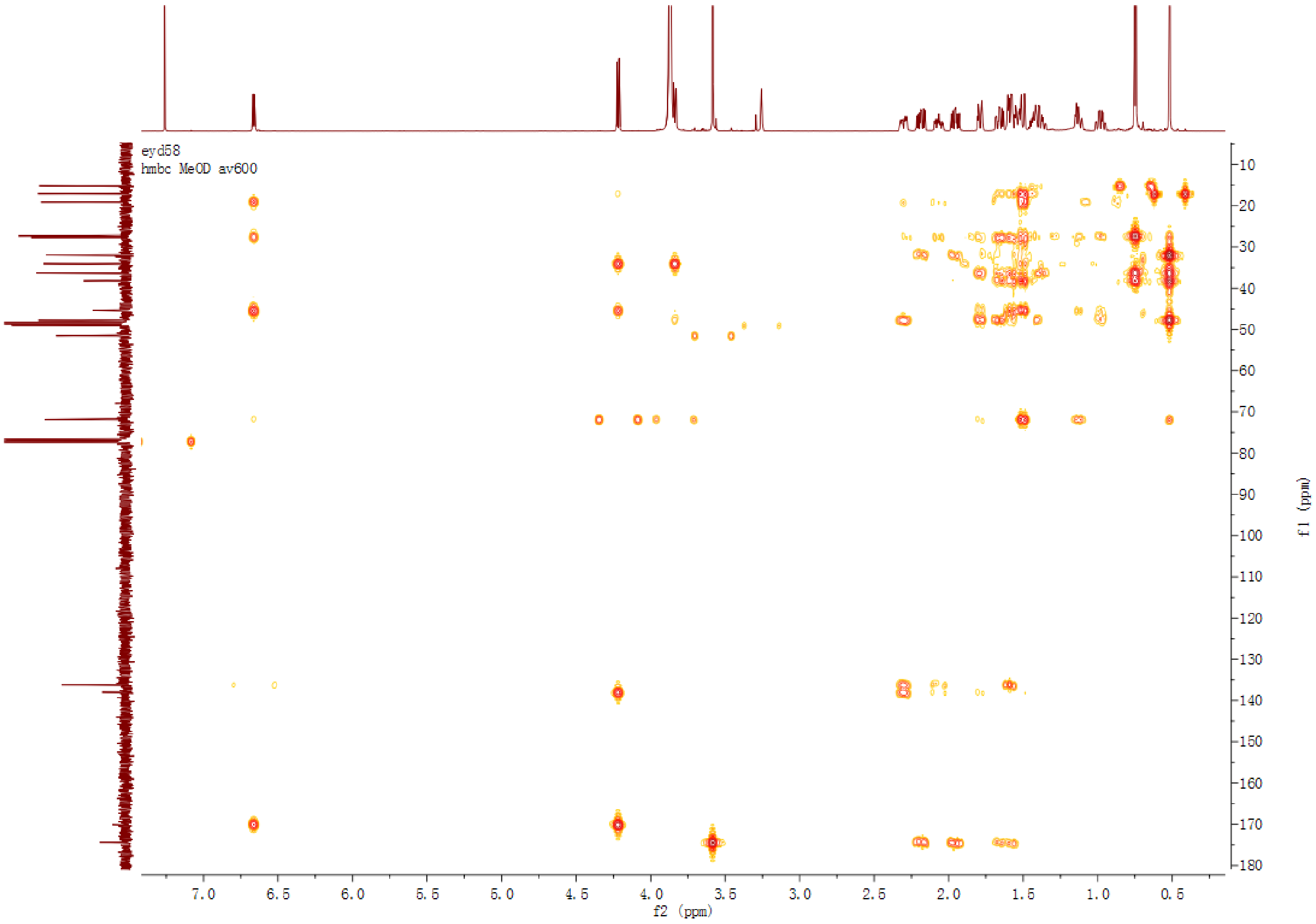

S21. ROESY spectrum (600 MHz, $CDCl_3$) of dodovislactone A (**5**).

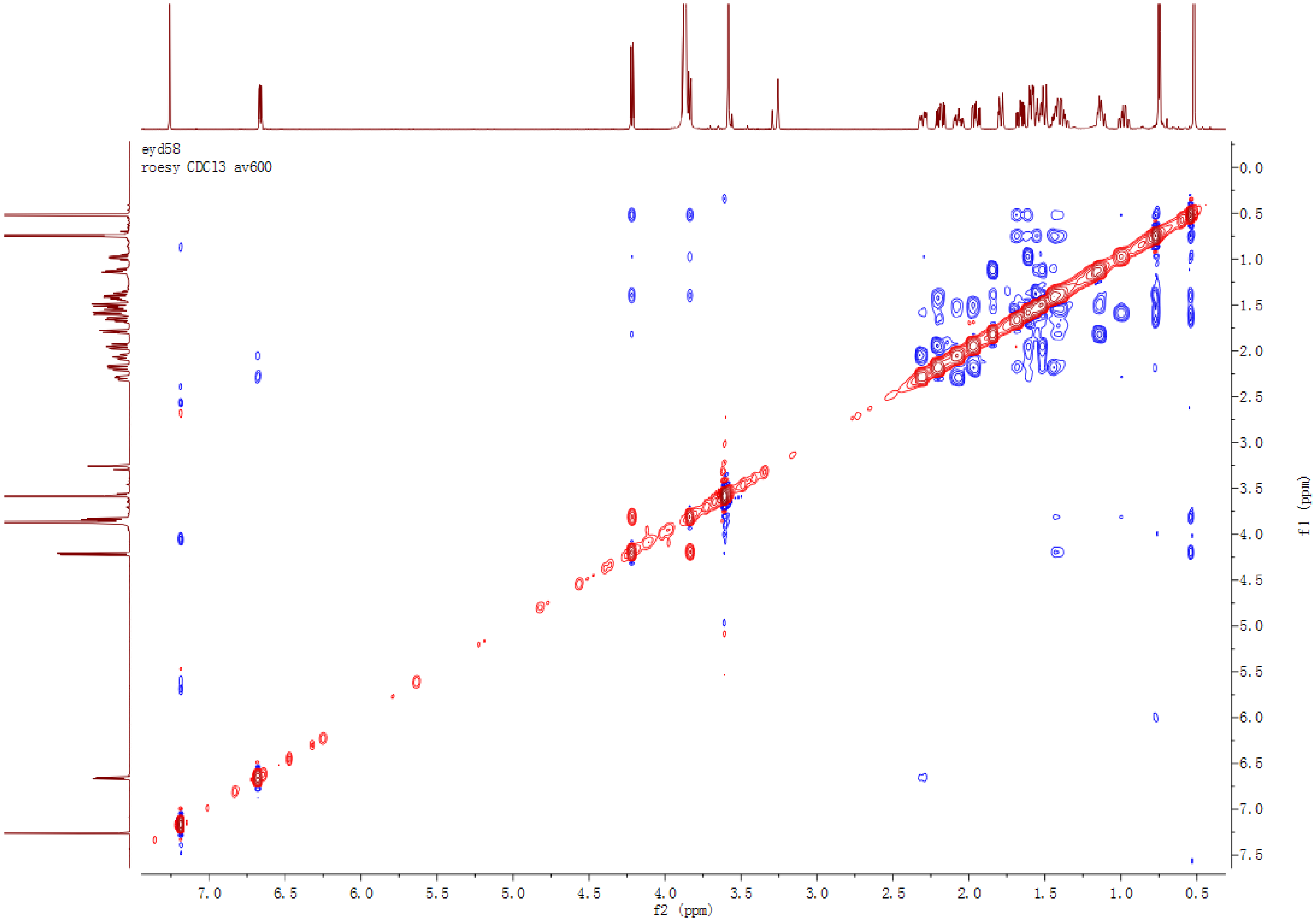

S22. UV spectrum (MeOH) of dodovislactone A (**5**).

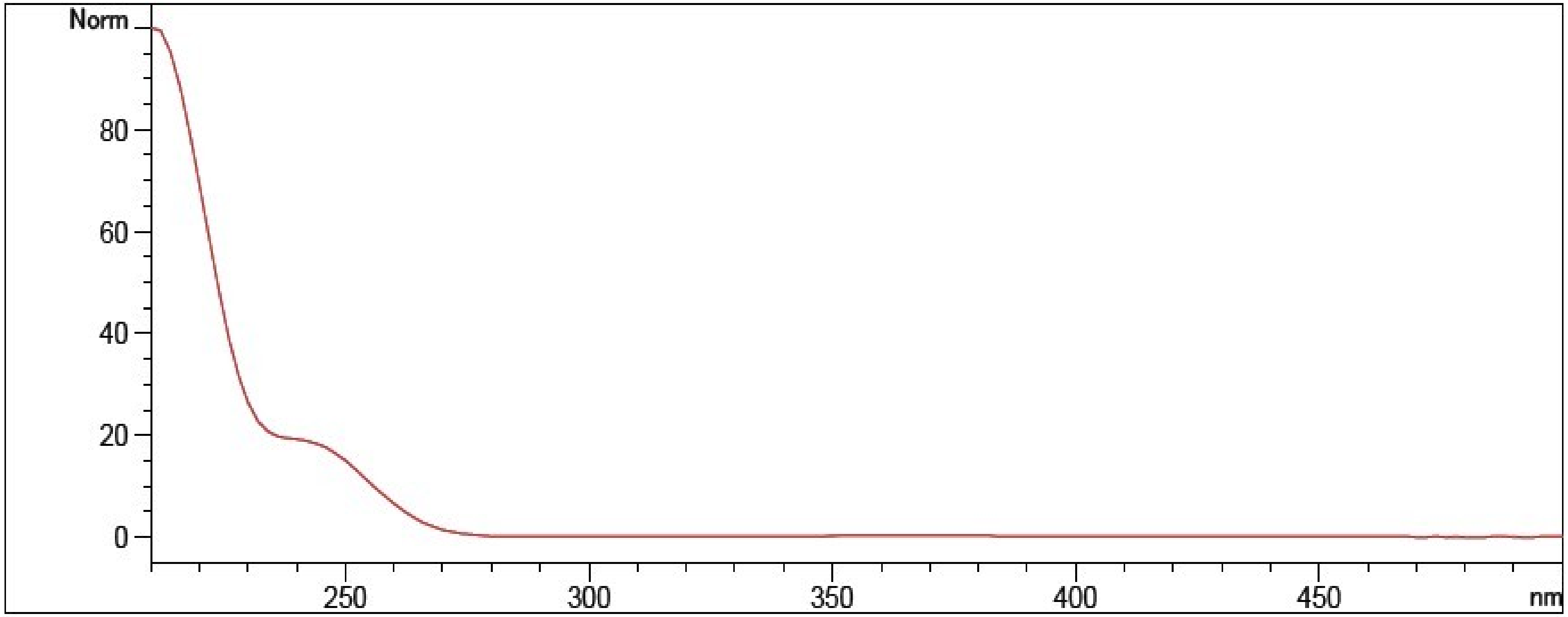

S23. ^{1}H NMR spectrum (600 MHz, $CDCl_3$) of dodovislactone B (**6**).

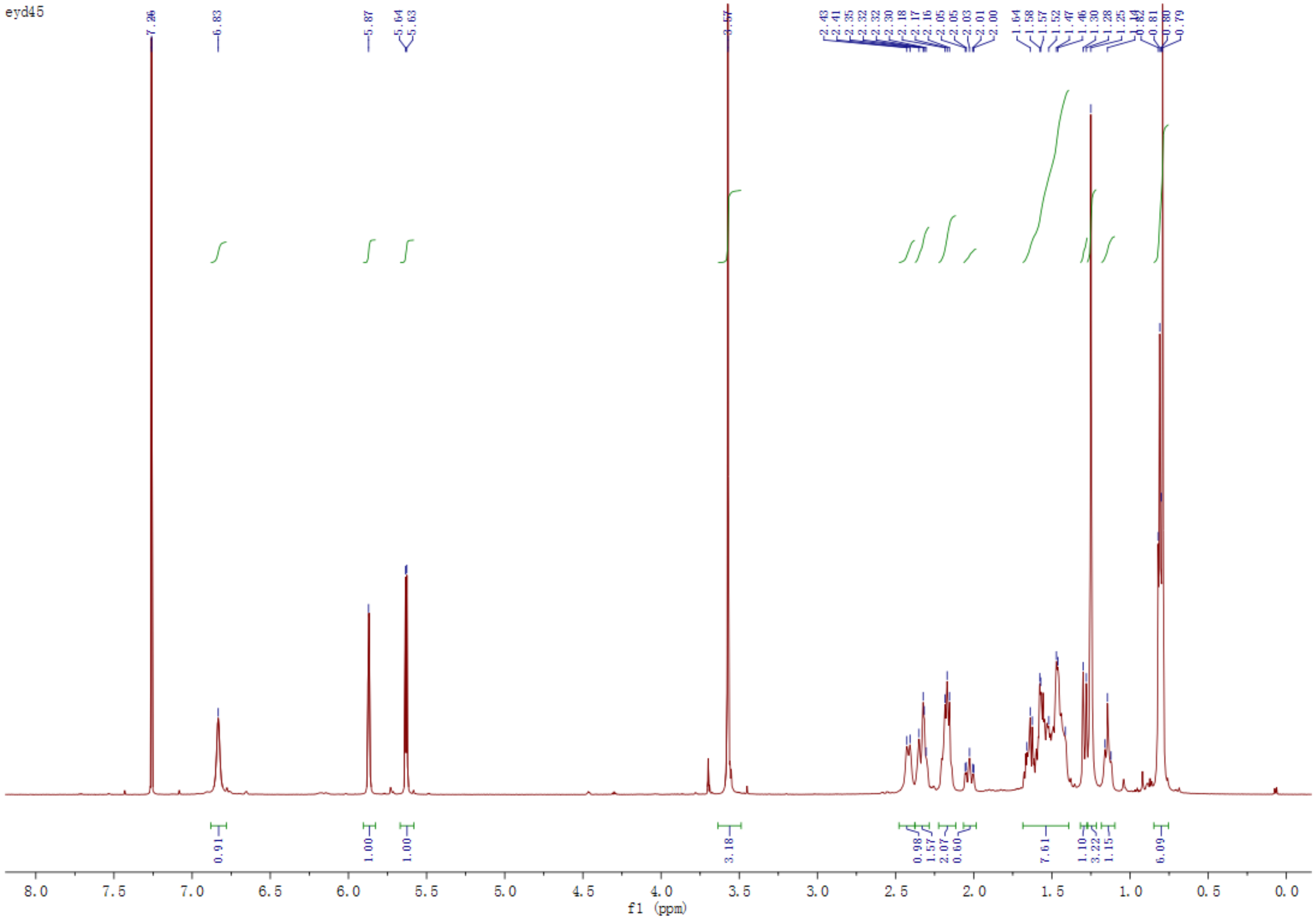

S24. ^{13}C NMR (DEPT) spectrum (150 MHz, $CDCl_3$) of dodovislactone B (**6**).

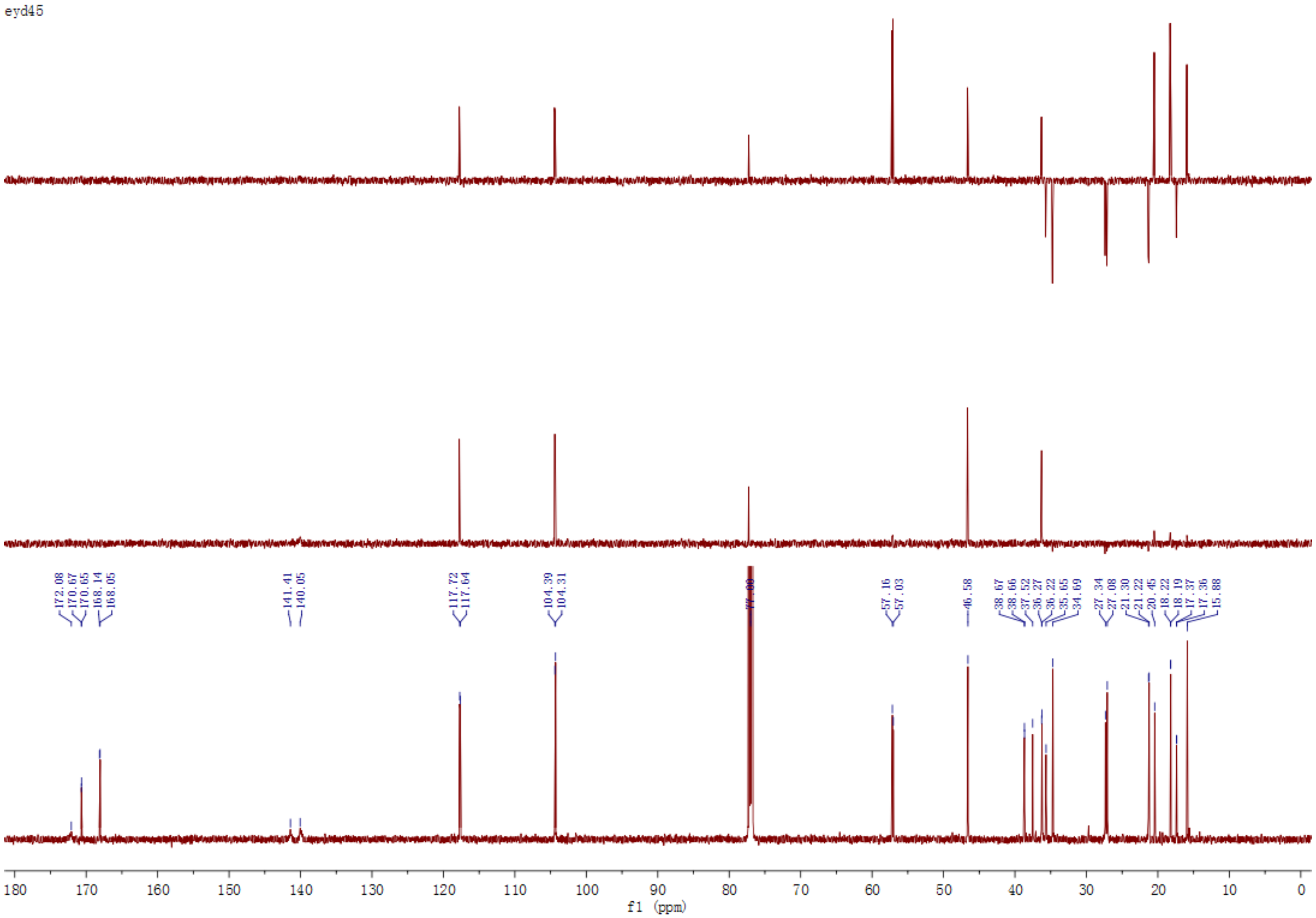

S25. HSQC spectrum (600 MHz, $CDCl_3$) of dodovislactone B (**6**).

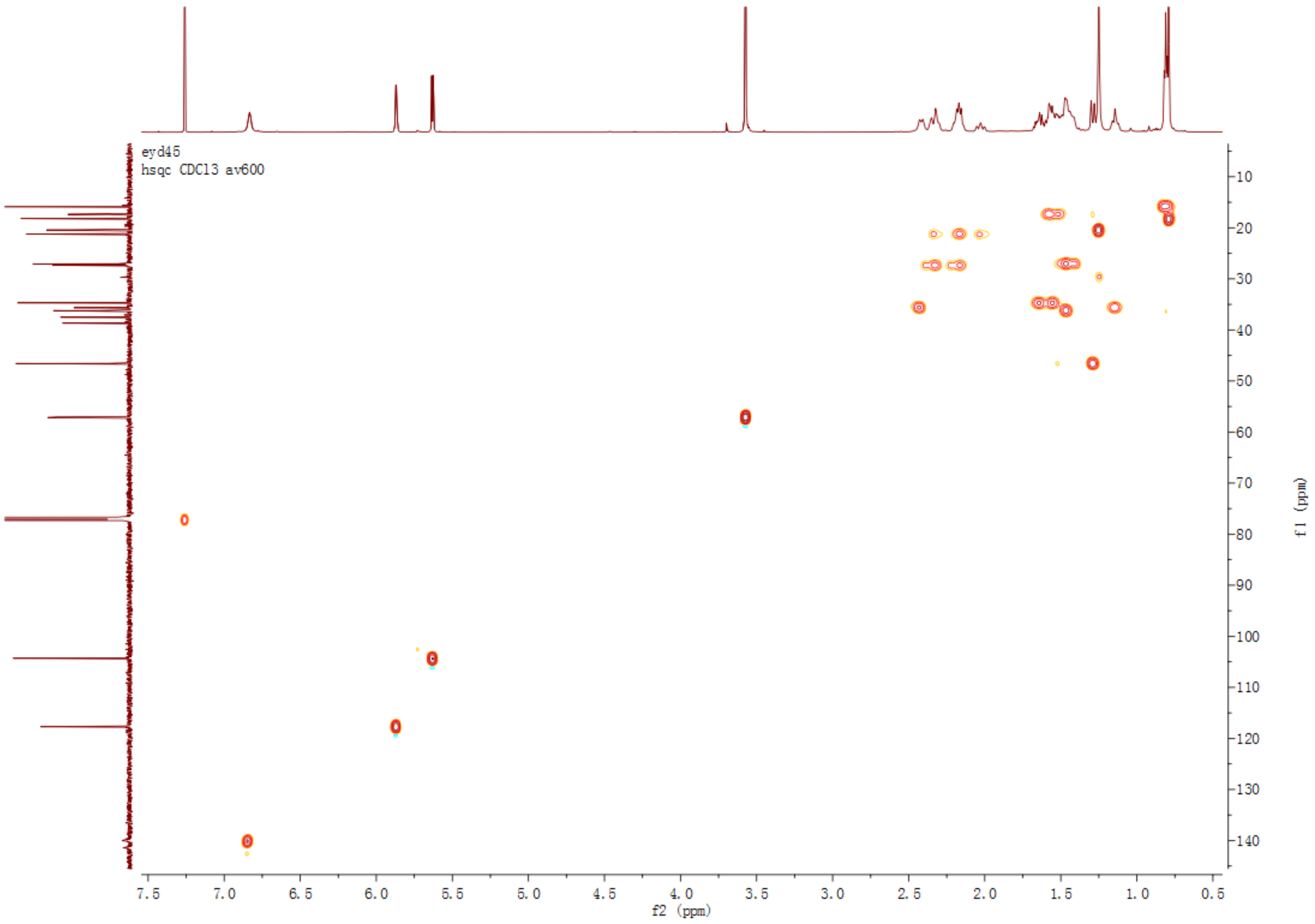

S26. HMBC spectrum (600 MHz, $CDCl_3$) of dodovislactone B (**6**).

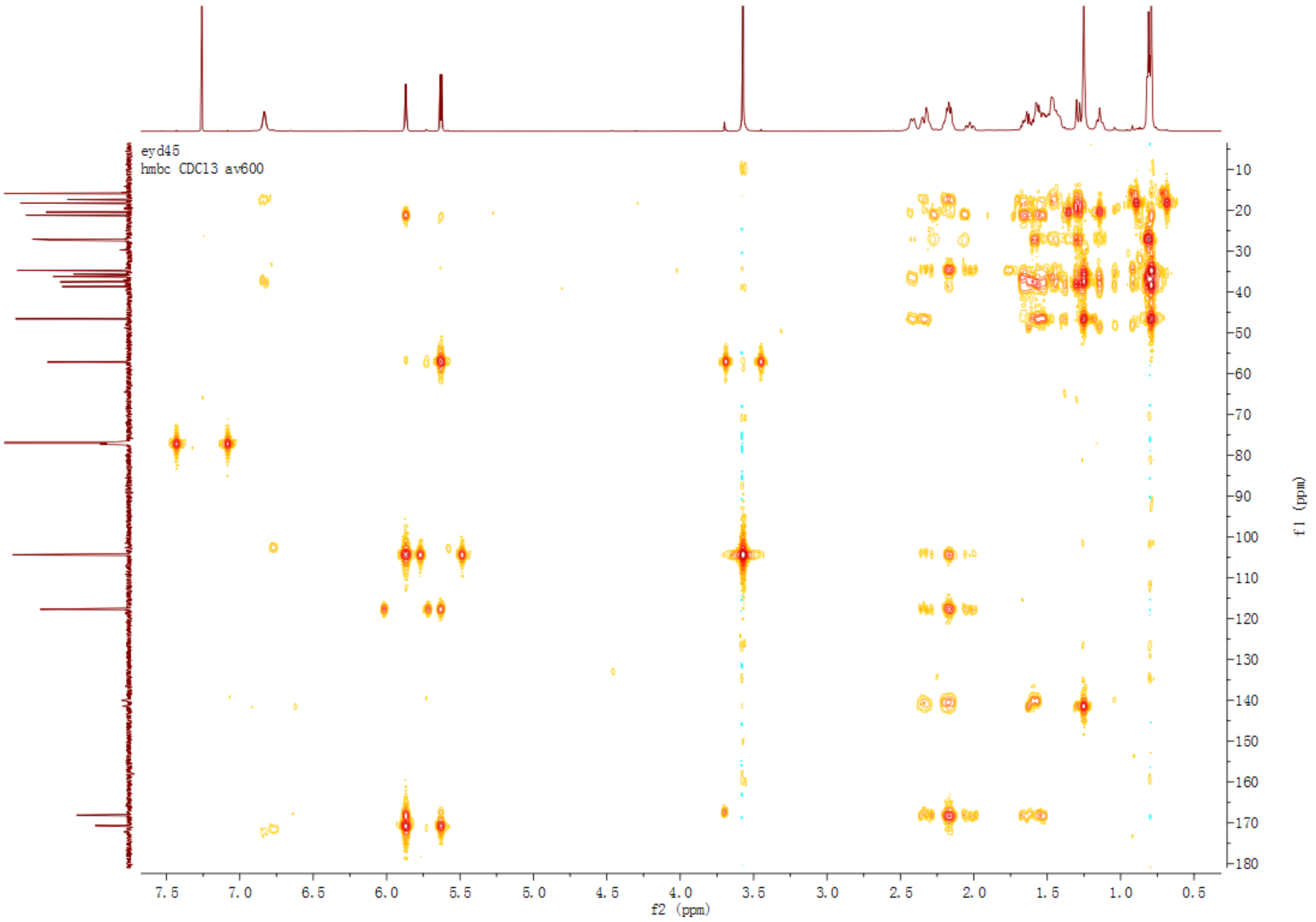

S27. ROESY spectrum (600 MHz, $CDCl_3$) of dodovislactone B (**6**).

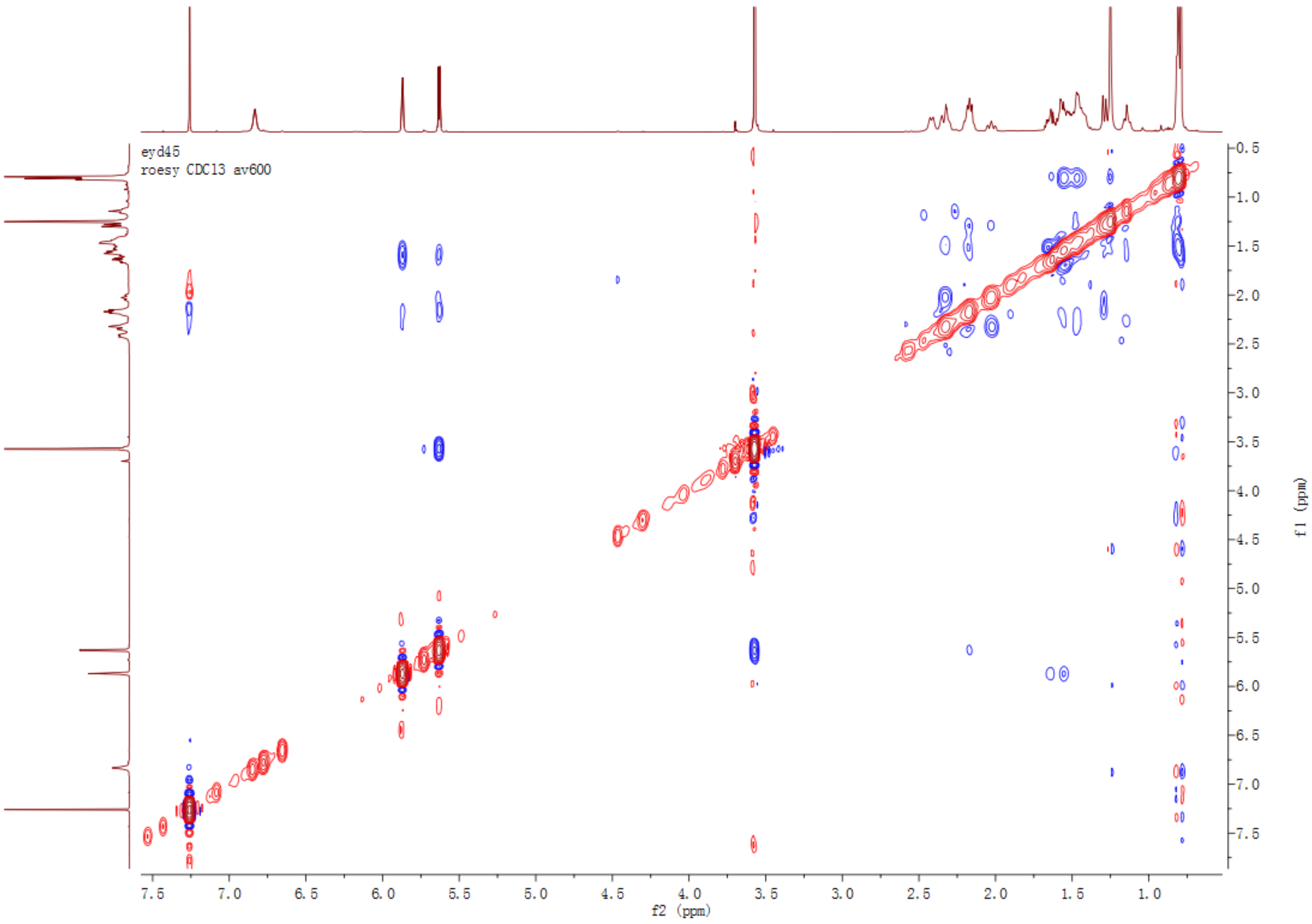

S28. UV spectrum (MeOH) of dodovislactone B (**6**).

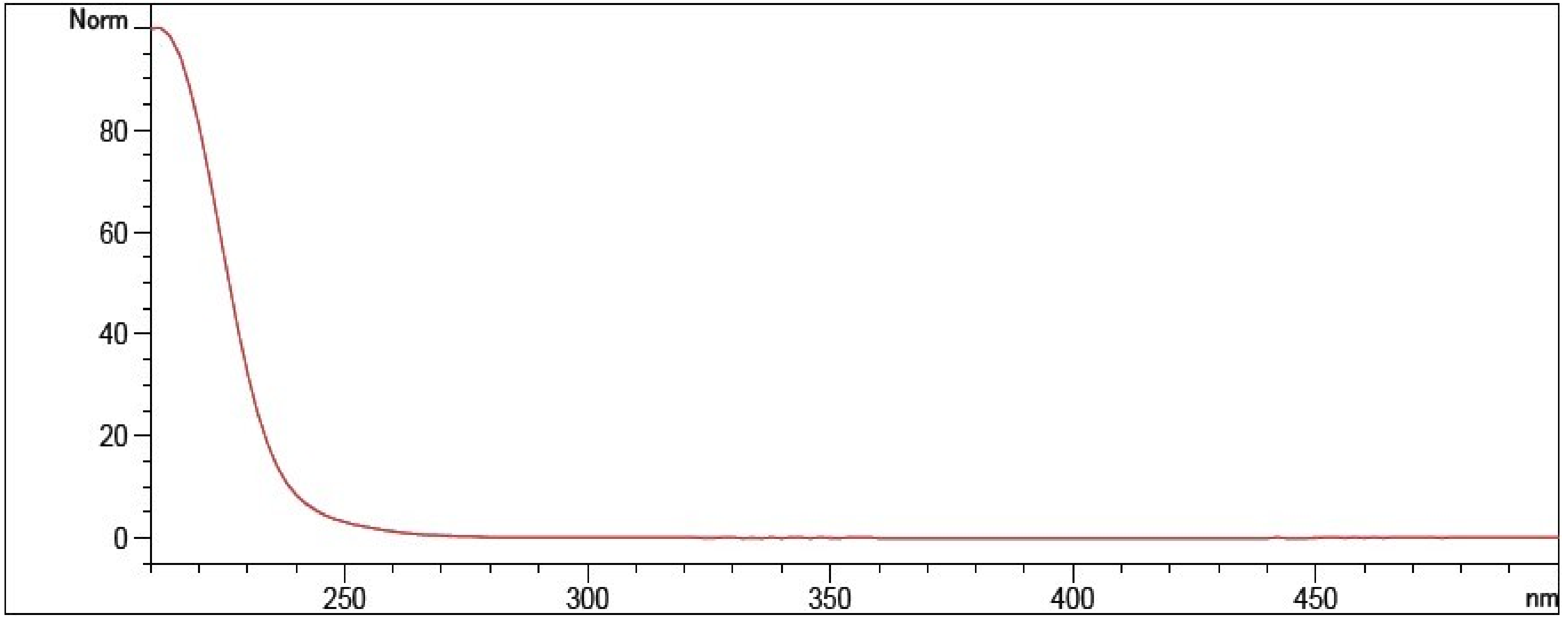

Supplement: Supplementary file 1 — Supplementary material, approximately 1.79 MB. [file 13659_2013_53_MOESM1_ESM.pdf]
